# Cannabidiol and brain function: current knowledge and future perspectives

**DOI:** 10.3389/fphar.2023.1328885

**Published:** 2024-01-15

**Authors:** Moniek Schouten, Sebastiaan Dalle, Dante Mantini, Katrien Koppo

**Affiliations:** ^1^ Exercise Physiology Research Group, Department of Movement Sciences, KU Leuven, Leuven, Belgium; ^2^ Movement Control and Neuroplasticity Research Group, Department of Movement Sciences, KU Leuven, Leuven, Belgium

**Keywords:** CBD, cannabis, hemp, endocannabinoid system, receptor, brain activity, neurological disease, mental disorder

## Abstract

Cannabidiol (CBD) is a naturally occurring non-psychoactive cannabinoid found in *Cannabis sativa*, commonly known as cannabis or hemp. Although currently available CBD products do not meet the safety standards of most food safety authorities to be approved as a dietary supplement or food additive, CBD has been gaining widespread attention in recent years due to its various potential health benefits. While primarily known for its therapeutic effects in managing epileptic seizures, psychosis, anxiety, (neuropathic) pain, and inflammation, CBD’s influence on brain function has also piqued the interest of researchers and individuals seeking to enhance cognitive performance. The primary objective of this review is to gather, synthesize, and consolidate scientifically proven evidence on the impact of CBD on brain function and its therapeutic significance in treating neurological and mental disorders. First, basic background information on CBD, including its biomolecular properties and mechanisms of action is presented. Next, evidence for CBD effects in the human brain is provided followed by a discussion on the potential implications of CBD as a neurotherapeutic agent. The potential effectiveness of CBD in reducing chronic pain is considered but also in reducing the symptoms of various brain disorders such as epilepsy, Alzheimer’s, Huntington’s and Parkinson’s disease. Additionally, the implications of using CBD to manage psychiatric conditions such as psychosis, anxiety and fear, depression, and substance use disorders are explored. An overview of the beneficial effects of CBD on aspects of human behavior, such as sleep, motor control, cognition and memory, is then provided. As CBD products remain largely unregulated, it is crucial to address the ethical concerns associated with their use, including product quality, consistency, and safety. Therefore, this review discusses the need for responsible research and regulation of CBD to ensure its safety and efficacy as a therapeutic agent for brain disorders or to stimulate behavioral and cognitive abilities of healthy individuals.

## 1 Introduction

### 1.1 Overview and statistics

Cannabidiol (CBD) is one of the over 140 types of cannabinoids found in *Cannabis sativa*. It is non-psychoactive and influences signaling pathways in the brain in a different way than tetrahydrocannabinol (THC), the key and most potent psychoactive compound of cannabis. THC is responsible for the “high” that is brought by the consumption of cannabis and is also responsible for dependence (Urits et al., 2021; Legare et al., 2022), whereas CBD is not intoxicating and does not induce abuse or dependence potential (Chesney et al., 2020; Urits et al., 2021). Furthermore, CBD is characterized by high lipophilicity and is therefore quickly distributed in the brain, adipose tissue, and other organs upon intake. However, it has low solubility and absorption in water when given in capsules, which causes variable pharmacokinetics. Bioavailability via inhalation averages 31%, while oral bioavailability is about 6% in humans (Chayasirisobhon, 2020).

The use of products derived from CBD is steadily increasing, especially in nations that have flexible regulations on cannabis-based products. However, even in countries where CBD products lack regulatory approval, they are still being sold and used to self-medicate against various conditions (Britch et al., 2021). CBD is mainly used to improve wellbeing and relieve symptoms of several diseases. Statistics on the prevalence of CBD as a therapeutic product globally are not widely available. The usage of CBD-based products as therapeutic agent is high in the United States and Canada, with 26.1% and 16.2%, respectively, whereas it is comparatively less popular in Europe, with a prevalence between 10.9% and 14% (Casanova et al., 2022; Goodman et al., 2022).

The results of an online survey to determine patterns of use, dose, and self-perceived effects of CBD showed that most users were aged between 25 and 54 years (72.2%) (Moltke and Hindocha, 2021). The most cited reasons for CBD use in this sample were anxiety (42.6%), sleep problems (42.5%), stress (37%), and general health and wellbeing (37%). Older people were more likely to use CBD for pain relief than any other reason, and CBD use was higher in males than in females. Respondents lauded the effectiveness of CBD and did not report adverse effects.

### 1.2 Importance of understanding the effects of CBD on brain function

There is increasing interest in the application of CBD as a therapeutic agent. Indeed, CBD has putative anticonvulsant, antipsychotic, anxiolytic, anti-inflammatory and anti-craving properties, which are critical in healthcare (Batalla et al., 2020). Since CBD has anticonvulsant properties, Epidiolex, the only FDA-approved prescription medicine based on CBD, is used worldwide to treat seizures associated with Lennox-Gastaut syndrome, Dravet syndrome and tuberous sclerosis complex. However, the low availability and affordability of antiseizure medications, especially in low-income countries, are the major barriers to epilepsy treatment (Leitinger et al., 2020). CBD may also be used to manage symptoms of anxiety and other mental disorders by regulating brain activity and connectivity patterns (O’Neill et al., 2021). Most conventional antipsychotics and antidepressants are linked to low response rates, adverse effects, limited tolerance, and adherence (Kruizinga et al., 2021). Mass production and distribution of CBD-based drugs might provide a solution for these challenges and could significantly increase the accessibility and adherence to psychiatric treatments while reducing adverse effects. However, knowledge gaps on the effects of CBD on brain function but also on the safety of CBD consumption first need to be addressed and a new regulatory pathway for CBD needs to be defined.

Understanding the effects of CBD on brain function has several advantages. First, it is important to establish the effectiveness of CBD-based products as an intervention for neurological and mental disorders, not to mention possible other applications in pain relief, sleep promotion, stress reduction, and cognitive enhancement. Second, investigating the effects of CBD on the brain is an opportunity to assess the risk to the brain posed by CBD, whether long-term or not. Finally, understanding whether CBD positively influences brain function has an implication for policymakers. As it stands, many nations are yet to legalize cannabis-based products, even for therapeutic purposes. Scientific evidence is crucial to drive policy change. If CBD demonstrates to be a viable intervention for any or all the named health issues, its use may improve the quality of life for millions of people globally. It is, therefore, important to review CBD’s effects on the brain using various scientific sources and methods.

### 1.3 Scope of the review

This review aims to gather, synthesize, analyze, and consolidate scientifically proven evidence on the effects of CBD on brain function and its significance in the treatment of brain disorders. To achieve this aim, some background information on CBD is provided, including its biomolecular properties and mechanisms of action. Next, the results emerging from human brain imaging and neurophysiological studies are discussed. Thereafter, the potential implications of CBD as a neurotherapeutic agent are addressed: the clinical applications of CBD in treating neurological and mental disorders are considered, as well as the applications of CBD to enhance aspects of human behavior. Finally, an overview of future research on CBD and its ethical implications is provided.

## 2 CBD and the endocannabinoid system

### 2.1 The endocannabinoid system (ECS) and its role in regulating brain function

The endocannabinoid system (ECS) controls most bodily functions, including sleep, temperature, pain reception, inflammatory and immune responses, learning and memory, processing emotions, and eating, making it the subject of most drug development research. The ECS has emerged as a mediator of short-term and long-term synaptic plasticity. It comprises at least two G protein-coupled receptors (GPCR), the accompanying endogenous ligands such as anandamide (AEA) and 2-arachidonoylglycerol (2-AG), and enzymes needed for synthesis and degradation (Lu and Mackie, 2016). The two major GPCRs are cannabinoid receptor type 1 (CB1) and cannabinoid receptor type 2 (CB2). The former is considered the most abundant GPCR in the central nervous system and is expressed widely across the prefrontal cortex, the hippocampus, and the basal ganglia (Freund et al., 2003; Harkany et al., 2008). CB2, on the other hand, is more prevalent immune cells.

The term “extended endocannabinoid system” is used to refer to a broader network of signaling molecules and receptors that interact with the ECS (Cristino et al., 2020). This is involved in the regulation of various physiological processes, including pain, inflammation, metabolism, and cardiovascular function. This expanded system includes other lipid signaling molecules such as N-acylethanolamines (NAEs) different than AEA that can activate cannabinoid receptors, as well as other receptors and enzymes that are involved in the regulation of various physiological processes. For example, NAEs such as palmitoylethanolamide (PEA) and oleoylethanolamide (OEA) can activate CB1 and CB2, as well as other receptors such as peroxisome proliferator-activated receptor alpha (PPARα) and transient receptor potential vanilloid channel 1 (TRPV1), which are involved in the regulation of pain and inflammation (Kasatkina et al., 2021). Similarly, 2-AG can activate both CB1 and CB2, as well as other receptors such as G protein-coupled receptor 55 (GPR55), which is involved in the regulation of blood pressure and inflammation (Sugiura and Waku, 2000; Balenga et al., 2011).

The fact that CB1 receptors form homodimers and heterodimers with other GPCRs, including CB2 receptors and dopamine, opioid, serotonin, and orexin receptors, and that several heterodimers have been described as specific modulators of hippocampal function further supports the location-dependent modulation of cannabinoid signaling (Batalla et al., 2020; Cristino et al., 2020). Activations of the heterodimers lead to effects that oppose the effects of isolated CB1 receptor activation. This phenomenon, together with different G proteins that can bind CB1, explain the diverse nature of the CB1 action depending on the neighboring protein interaction and location.

The ECS controls the activity-based forms of plasticity by inducing long-term changes in the γ -aminobutyric acid (GABA) release (Winters and Vaughan, 2021). Hippocampal excitation can induce the production of 2-AG, which then acts on the presynaptic GABAergic synapsis, thereby modulating excitability by regulating the inhibitory tone of hippocampal circuits (Abate et al., 2021). Further, synaptic plasticity in the amygdala is critical in the acquisition, storage, and extinction of aversive memories, and the ECS has emerged as a crucial mediator of such neuroplasticity-related phenomena (Viveros et al., 2007). It was proposed that endocannabinoids facilitate the extinction of aversive memories through their selective inhibitory effects on local inhibitory networks in the amygdala. This provides evidence for the functional role of endocannabinoid release-based synaptic plasticity (Marsicano et al., 2002; Azad et al., 2004). In addition to the amygdala, the hypothalamus has been suggested as a potential site for cannabinoid-induced neural plasticity. In this area, cannabinoid-dependent synaptic plasticity is believed to play a role in regulating the stress-response system (Di et al., 2003; Riedel and Davies, 2005).

### 2.2 CBD-related signal transduction

**FIGURE 1:**
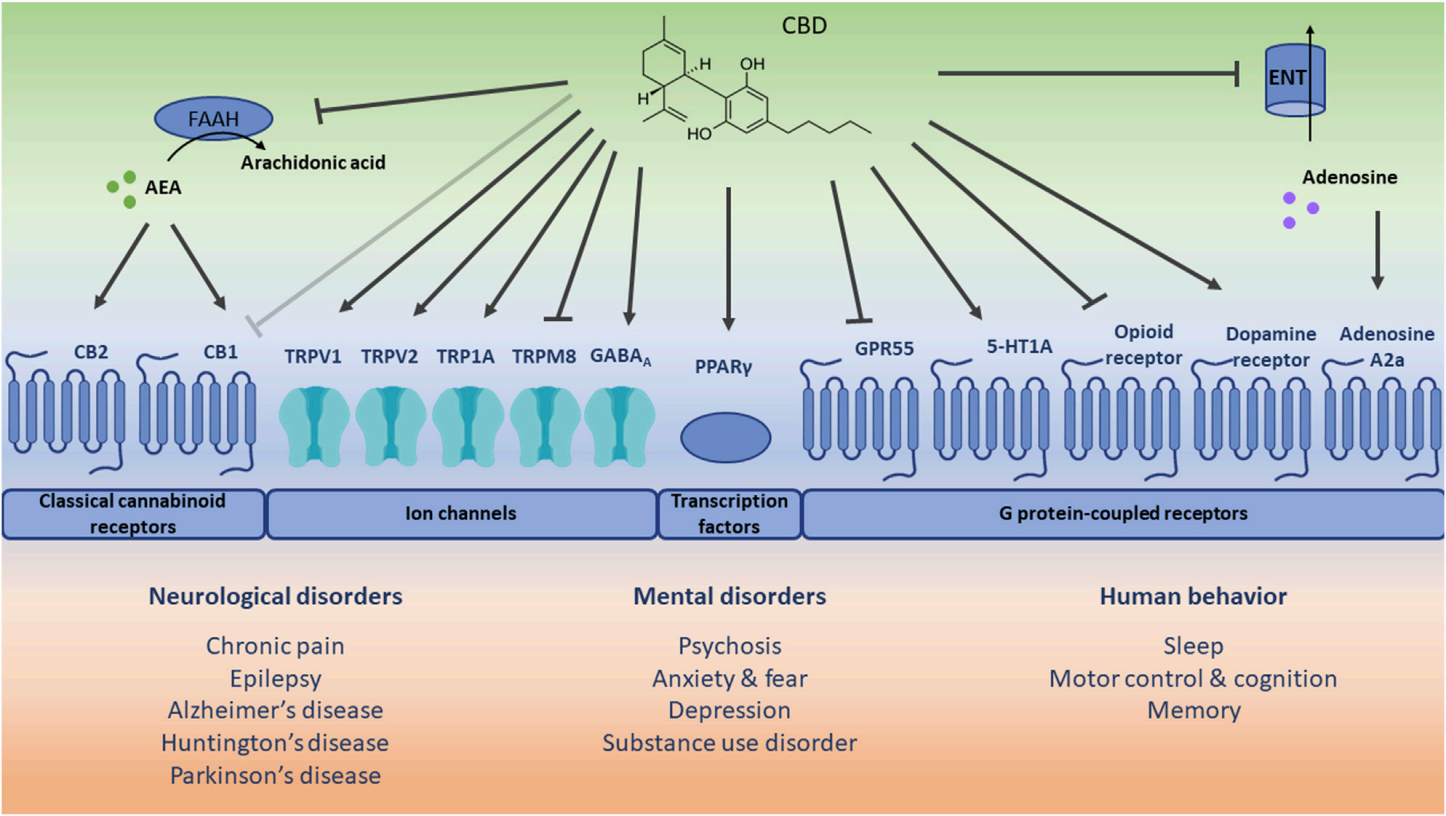
Schematic representation of potential receptor-dependent and independent mechanisms via which cannabidiol (CBD) may play a role in the treatment of therapeutic applications. The direct (ant)agonistic actions of CBD are depicted with arrows and lines toward the respective receptors. Additionally, the indirect receptor mechanisms of CBD, i.e., via increased anandamide (AEA) concentration [by inhibiting its hydrolysis to arachidonic acid via fatty acid amide hydrolase (FAAH)], and increased extracellular adenosine concentration [by inhibiting its uptake via equilibrative nucleoside transporter (ENT)] are included. The light grey inhibitory arrow indicates an antagonistic effect of CBD towards cannabinoid receptor 1 (CB1). However, it should be noted that CBD displays a low affinity for CB1 and a minimal direct activity at CB1. CB2: cannabinoid receptor 2; TRPV1: transient receptor potential vanilloid channel 1; TRPV2: transient receptor potential vanilloid channel 2; TRPA1: transient receptor potential ankyrin channel 1; TRPM8: transient receptor potential melastin channel 8; GABA_A_: γ-aminobutyric acid type A receptor; PPARγ: peroxisome proliferator-activated receptor gamma; GPR55: G protein-coupled receptor 55; 5-HT1A: serotonin 1A receptor.

Although the molecular pathways and mechanisms through which CBD acts have not been fully established yet (Batalla et al., 2020), it is suggested that CBD can directly interact with different receptor-dependent and independent mechanisms (Schouten et al., 2022), thereby targeting multiple pathways and mechanisms of action which contribute to different therapeutic applications (Britch et al., 2021; Legare et al., 2022) (see Figure 1). CBD engages in the activation of channels (e.g., TRPV1), transcription factors (e.g., PPARγ), and different GPCRs, such as CB1, CB2, GPR55, serotonin 1A receptor (5-HT1A), and adenosine A2a receptor.

TRPV1 regulates the transduction of (noxious) chemical and physical stimuli such as acid, capsaicin (hot pepper), allyl isothiocyanate (wasabi), and heat, but also of endocannabinoids. Upon activation, desensitization of the receptor might occur, which relieves the symptoms of nociceptive behavior. CBD acts as a TRPV1 agonist and can thus induce receptor desensitization resulting in analgesic effects, as was observed in animal models of neuropathic and inflammatory pain (Costa et al., 2004). Less is known about the effect of CBD on TRP1A (a sensor of cold, pain, and itch) and TRPV2 (a high-threshold thermosensor), though it was found that CBD also activates and desensitizes these receptors, which was reported to play a role in neuronal inflammation (Wang et al., 2019) and development (Santoni and Amantini, 2019). In contrast, CBD is an antagonist of the cold and menthol receptor (TRPM8), which is also expressed in sensory neurons, but the functional role of CBD on TRPM8 remains to be further elucidated. It was recently also shown that CBD inhibits voltage-gated sodium channels (Huang et al., 2023) and activates the voltage-gated potassium channel KCNQ2 (Ma et al., 2023), which both play a major role in regulating neuronal excitability.

As mentioned before, CBD does not only exert its action via the activation of channels but also via transcription factors such as PPARγ which is engaged in metabolic and immune functions. As an agonist of PPARγ, CBD was shown to exert anti-inflammatory (Hou et al., 2012) and neuroprotective (Esposito et al., 2011) effects.

Due to its low affinity for CB1 and CB2 (Bisogno et al., 2001), CBD mainly mediates CB1 and CB2-related signaling via indirect ways. CBD inhibits the enzymatic activity of fatty acid amide hydrolase which is responsible for the degradation of AEA. Since AEA also acts as an agonist of the cannabinoid receptors, CBD and its related increase in AEA abundance might therefore indirectly stimulate CB1 and CB2 signaling. However, CBD might also attenuate binding of CB1 agonists, thereby antagonizing CB1 signaling (Laprairie et al., 2015). In the CNS, CBD-related effects on mainly CB1 and CB2 are involved in the regulation of neuropathic and nociceptive pain but also anxiety. Additionally, CB2 is highly expressed in immune cells, and agonism of CB2 induces anti-inflammatory effects. CBD is also an antagonist of GPR55, via which it might exert anti-inflammatory effects (Lin et al., 2011). Furthermore, CBD may act via 5-HT1A which plays a protective role in oxidative stress and more specifically lipid peroxidation. In an animal model of hypoxic-ischemic brain injury, CBD treatment partially prevented oxidative stress, inflammation and excitotoxicity via 5-HT1A (Pazos et al., 2013). Finally, CBD also affects the adenosine A2a receptor via an indirect mechanism, i.e., CBD inhibits the equilibrative nucleoside transporter, which controls extracellular adenosine availability for other cell receptors. The accumulation of extracellular adenosine levels eventually stimulates the adenosine A2a receptor via which CBD might exert anti-inflammatory (Carrier et al., 2006) and neuroprotective (Castillo et al., 2010) effects.

There is also evidence that CBD affects the binding capacity of ligands to receptors of the opioid and dopaminergic system. CBD decreases the binding capacity of agonists to µ- and δ-opioid receptors, thereby downregulating opioid receptor signaling (Kathmann et al., 2006). This might hold clues for CBD treatment as a strategy for addictions (Hurd et al., 2015), such as alcohol misuse (Viudez-Martínez et al., 2018a). CBD also acts as a partial agonist of dopamine D2 receptors, which might have antipsychotic effects (Seeman, 2016). Finally, via facilitated binding of agonists to GABA type A (GABA_A_) receptors, CBD can be relevant for anticonvulsant and anxiolytic effects (Bakas et al., 2017). Although promising, it should be noted that these relationships and therapeutic applications require further investigation.

## 3 Evidence for CBD effects in the human brain

Human neuroscience techniques such as magnetic resonance imaging (MRI), positron emission tomography (PET), single-photon emission computed tomography (SPECT), and electroencephalography (EEG) can be used to shed light and guide the use of CBD. It has been established that depending on the region and dose, CBD can elicit different reactions (Millar et al., 2018). Brain imaging helps to understand which dose of CBD elicits what kind of reaction in a specific brain region (Weinstein et al., 2016). Nonetheless, these brain regions are interconnected, and the effects of CBD on one region can have implications for the functioning of others (Lorenzetti et al., 2023). It is worth noting that research in this field is still ongoing, and there is much to learn about the interaction between CBD and the brain.

### 3.1 Magnetic resonance imaging

Several functional MRI (fMRI) studies investigated the effects of CBD on brain function in healthy individuals (Supplementary Table S1). Overall, CBD was found to reduce resting-state activity and connectivity across several brain regions, potentially indicating an anxiolytic (anxiety-reducing) effect. In a cross-over study using fMRI, sixteen healthy volunteers were given 600 mg of oral CBD (Grimm et al., 2018). The group that was given CBD significantly increased the fronto-striatal connectivity compared to the placebo group. THC, on the other hand, did not show a significant change in connectivity. A direct comparison between THC and CBD revealed that CBD increased connectivity relative to THC between the right putamen and the frontal pole and paracingulate gyrus. In response to inhibition, CBD was found to attenuate brain activity in the left posterior insula, left superior temporal gyrus, and left transverse temporal gyrus. The left medial prefrontal cortex was attenuated by CBD during the presentation of salient stimuli relative to non-salient stimuli (Borgwardt et al., 2008). In addition, CBD increased the connectivity between the left caudate nucleus, the left inferior frontal gyrus, and the left dorsal striatum. In contrast, it decreased connectivity between the left dorsal striatum, the left anterior cingulate, and the left thalamus.

Other studies reported that CBD may have a modulatory effect on brain regions associated with anxiety and stress, such as the amygdala and prefrontal cortex. For instance, a study documented that, during emotional processing, CBD attenuated bilateral activity in the posterior lobe of the cerebellum (Batalla et al., 2020). In intense fear, CBD attenuated activity in the amygdala, anterior parahippocampal gyrus, anterior and posterior cingulate gyri, and the right posterior lobe of the cerebellum (Batalla et al., 2020), and disrupted the connectivity between the left amygdala and the left anterior cingulate cortex when processing fear. Also, CBD increased activity in the right occipital lobe, lingual gyrus, cerebellum, and cuneus during visual stimulation (Winton-Brown et al., 2011).

An fMRI study compared brain activation during reward, loss, and neutral anticipation in medication-naïve individuals with high risk for psychosis (Wilson et al., 2019). The group given CBD showed intermediate activation in the left insula and parietal operculum, left superior frontal gyrus, and left frontal operculum. In patients with established psychosis, CBD treatment showed bilateral activation in the inferior frontal and left middle frontal gyrus (Fusar-Poli, 2012). In patients with anxiety disorder, fMRI studies reported the activation of the right posterior cingulate gyrus (Li et al., 2018), while activity was decreased in the left parahippocampal gyrus and hippocampus (Cha et al., 2016). Finally, an MR spectroscopy study showed that CBD decreased GABA in patients with autism but not in the healthy control groups (Pretzsch et al., 2019).

### 3.2 Nuclear medicine imaging

PET and SPECT studies have been less common in investigating the effects of CBD compared to MRI studies. Some of these studies have explored the binding of CBD to specific receptors in the brain, such as the serotonin 5-HT1A receptor, which is associated with mood regulation.

A study used PET to investigate the effects of CBD on brain function in people at high risk of psychosis (Bhattacharyya et al., 2018). The administration of 600 mg of CBD was found to be associated with changes in brain function in the medial temporal, midbrain, and striatal regions, which are areas that are involved in the development of psychosis. These changes were associated with improvements in cognitive performance and reduced symptoms of psychosis. The findings corroborate the idea that CBD may have potential as a treatment for people at high risk of developing psychosis. A case report study provided further evidence for the positive effects of CBD in psychosis (Koethe et al., 2023), suggesting that these may be due to enhanced cerebral glucose utilization.

Other studies on the neural effects of CBD were conducted using SPECT. Specifically, in two SPECT studies healthy individuals (Crippa et al., 2004) and treatment-naïve patients with social anxiety disorder (Crippa et al., 2011) were administered with 400 mg of CBD or placebo, in a double-blind procedure. CBD significantly decreased subjective anxiety and increased mental sedation, while placebo did not induce significant changes in both studies. In healthy individuals, lower activity in CBD than placebo was revealed in the left amygdala and the left posterior cingulate gyrus. In patients with social anxiety disorder, CBD administration reduced activity in the left parahippocampal gyrus, hippocampus, and inferior temporal gyrus, relative to placebo. Taken together, these results suggest that CBD can reduce anxiety, and that this is related to its effects on activity in limbic and paralimbic brain areas.

### 3.3 Electroencephalography

Evidence for the effects of CBD on EEG activity in healthy individuals is currently lacking. EEG recordings were mainly used to document the effects of CBD on patients with seizures. For instance, a case study on a nine-year-old with Lennox-Gastaut Syndrome, a severe and rare form of epilepsy, documented that CBD produced an unprecedented dramatic normalization of the baseline EEG activity (Prakash, 2020). More generally, a meta-analysis of 104 EEG studies from 52 patients with pediatric-onset refractory epilepsy reported that 74% of the patients had a reduction in interictal epileptiform discharges (IEDs), 46% in ictal findings, and 17% experienced a change in sleep architecture after CBD treatment (Herlopian et al., 2022). Another study assessed the longitudinal impact of CBD on EEG measures in subjects with treatment-resistant epilepsy (Grayson et al., 2021). It showed that CBD has positive effects on interictal epileptiform discharge frequency but no effects on other clinical EEG measures; the effects of CBD did not appear to be dependent on dose. Another longitudinal EEG study in patients with refractory epilepsy (Armstrong et al., 2022) revealed that there are subtle changes in certain neural metrics even at baseline that may not be perceived during qualitative analysis and that could be used in the future as a biomarker to predict a patient’s clinical response to CBD administration.

## 4 Implications for the potential of CBD as a therapeutic agent

The following sections review clinical and pre-clinical data concerning the therapeutic potential of CBD in a variety of neurological and mental disorders as well as its effects on aspects of human behavior (Supplementary Table S2). However, in some disorders clinical trials are scarce and conclusions are predominantly based on preclinical studies, highlighting the need for human intervention trials.

### 4.1 Neurological disorders

#### 4.1.1 Chronic pain

Pain lasting longer than 3 months can be classified as chronic pain, and involves neuropathic, nociceptive, musculoskeletal, inflammatory, psychogenic, and mechanical pain (Cohen et al., 2021).

Preclinical studies show great potential for CBD as an analgesic agent (Costa et al., 2007; Belardo et al., 2019; De Gregorio et al., 2019; Jesus et al., 2019; Malvestio et al., 2021). The analgesic properties of CBD are suggested to be induced by the agonistic actions of CBD at TRPV1, a mediator in pain signaling (Henson et al., 2022). Once TRPV1 channels are activated, they desensitize and enter a refractory period during which sensory neurons are not responsive to further stimulation. Via this mechanism, symptoms of nociceptive behaviour are relieved. Indeed, rodent models of neuropathic and inflammatory pain showed that the antihyperalgesic actions of CBD were reversed by TRPV1 antagonists (Costa et al., 2004; Costa et al., 2007; De Gregorio et al., 2019). Additionally, CBD’s analgesic actions may be mediated via the activation of the serotonergic system through 5-HT1A receptors. In male diabetic rats, mechanical allodynia was attenuated by treatment with CBD (0.3 or 3 mg/kg). This effect was completely prevented by the pre-treatment with the 5-HT1A antagonist WAY 100135 (Jesus et al., 2019). Furthermore, in rats subjected to a nerve injury model that induces neuropathic pain, 5-HT firing activity decreased, resulting in mechanical allodynia. Treatment with CBD (5 mg/kg/d) normalized 5-HT activity and reduced mechanical allodynia. However, after antagonism of 5-HT1A with WAY 100635 (2 mg/kg/d for 7 days) this effect was partially prevented (De Gregorio et al., 2019). These findings are in accordance with a similar study in rats subjected to a nerve injury model, which also showed that treatment with CBD (30 nmol microinjected into the prelimbic division of the medial prefrontal cortex) attenuated mechanical allodynia in a 5-HT1A-dependent manner (Malvestio et al., 2021). Interestingly, it was found that the analgesic effect of CBD was also blocked by the CB1 receptor antagonist, providing an alternative mechanism of action (Malvestio et al., 2021).

Although these preclinical studies repeatedly show the analgesic properties of CBD, evidence in human clinical trials remains scarce. A survey investigating patients’ perspectives and attitudes about CBD for the treatment of pain symptoms showed that 62% of the participants used a CBD product. Furthermore, the majority of patients (59%) who ever used CBD products perceived reductions in pain and in 67.6% of these patients, the use of CBD allowed them to reduce their pain medications (Schilling et al., 2021). Another explorative study evaluated the effect of CBD treatment on self-reported quality of life and showed that patients with (non-)cancer chronic pain symptoms reported reductions in pain scores upon CBD treatment, while patients with neurological symptoms did not perceive any improvements (Gulbransen et al., 2020). The lack of a control group in this study makes it difficult to identify any causal relationship for CBD treatment and reduced pain scores. Additionally, there was a large loss of participants (36.3%) during the study. Furthermore, the applied doses ranged from 40 to 300 mg/d and were reported inconsistently and incompletely by patients, which further added complexity to the interpretation of these results.

In a study involving seven kidney transplant patients with chronic pain, CBD treatment (100–300 mg/d for 3 weeks) resulted in total pain reduction in two patients and a partial reduction in four patients, while one patient did not perceive any change in symptoms at all (Cuñetti et al., 2018). Again, the lack of a control group and the low sample size make it impossible to draw any conclusions from this study, as acknowledged by the authors. On the other hand, a placebo-controlled randomized trial involving 29 patients with symptomatic peripheral neuropathy provided evidence that transdermal application of CBD oil (250 mg/3 fL. Oz for 4 weeks) can yield a significant reduction in intense pain, sharp pain, cold and itchy sensations compared to placebo (Xu et al., 2020).

To date, clinical studies on the effect of CBD in patients with chronic pain remain inconclusive. The majority of clinical studies for the treatment of chronic pain typically utilized a mixture of THC and CBD [reviewed in (Henson et al., 2022; Argueta et al., 2020)] and studies evaluating the isolated effect of CBD are rather scarce and unconvincing. As such, there is a high need for placebo-controlled clinical studies evaluating the effect of isolated CBD supplements to clarify the role and the underlying mechanisms of CBD in chronic pain management.

#### 4.1.2 Epilepsy

Epilepsy is a neurological disease characterized by cerebral hyperactivity or synchronous neuronal activity (Fisher et al., 2005). A large proportion of epileptic patients suffer from drug-resistant seizures that further increase the rate of cognitive impairment as well as psychiatric and physical disability.

CBD has been shown to exert anti-convulsant effects in various animal models of epileptic seizures (Jones et al., 2012; Klein et al., 2017; Costa et al., 2022). It is hypothesized that CBD may act preferentially to reduce seizure spread (Jones et al., 2012), but the exact mechanism underlying the anti-convulsant effects of CBD has not yet been elucidated. Given its low affinity for CB1 and CB2, CBD most likely modulates neuronal hyperexcitability via cannabinoid receptor-independent pathways. Proposed mechanisms include: 1) regulation of Ca^2+^ homeostasis in neurons under a normal or a highly excitable state (Ryan et al., 2009); 2) agonistic properties at 5-HT1A receptors eliciting membrane hyperpolarising responses (Theodore, 2003; Merlet et al., 2004); 3) enhancing endogenous adenosine levels in the CNS thereby increasing inhibitory adenosinergic tone which aids in seizure suppression (Boison, 2006; Carrier et al., 2006); and 4) inducing neuroprotective and anti-inflammatory actions via modulation of PPARγ (Costa et al., 2022). It is critical to understand these mechanisms of action to improve CBD’s efficacy and implementation as a therapeutic agent in epilepsy.

Also in human clinical trials, the potential of CBD as a therapeutic agent for drug-resistant seizures has recently been investigated. Its effectiveness in childhood epilepsy including, Lennox–Gastaut syndrome and Dravet syndrome has been studied extensively and summarized in recent systematic reviews (da Silva Rodrigues et al., 2023; Silvinato et al., 2022). It was concluded that treatment with CBD successfully reduced the frequency of seizures by 33%, supporting the use of CBD in these patient populations. These positive results have led to the approval of Epidiolex as a prescription medicine for seizures associated with Lennox–Gastaut syndrome and Dravet syndrome.

#### 4.1.3 Alzheimer’s disease

Alzheimer’s disease (AD) is a neurodegenerative disease that is characterized by parenchymal deposition of amyloid-β in plaques and intraneuronal accumulation of hyperphosphorylated tau protein, inducing chronic inflammation and oxidative damage (DeTure and Dickson, 2019). Several studies showed a potential role for CBD in the inhibition of AD progression [reviewed in (Xiong and Lim, 2021)].

Treatment with CBD was reported to reduce amyloid-β production, tau phosphorylation, and neuroinflammation *in vitro* (Scuderi et al., 2014; Libro et al., 2016). These effects were also observed in *in vivo* studies where CBD treatment suppressed the neuroinflammatory response induced by amyloid-β deposition in mice (Esposito et al., 2007) and rats (Esposito et al., 2011), and stimulated hippocampal neurogenesis (Esposito et al., 2011) thereby delaying disease progression. CBD also improved cognitive performance, which is typically affected in AD. More specifically, long-term treatment with CBD (20 mg/kg/d for 8 months) prevented the development of social recognition deficit in a transgenic mouse model of AD (Cheng et al., 2014a). Additionally, sub-chronic treatment with CBD (20 mg/kg daily for 1 week, 3x/week for the following 2 weeks) prevented the cognitive impairment in mice injected with amyloid-β as shown by their performance in the Morris water maze test (Martín-Moreno et al., 2011). These positive effects on cognitive functioning were associated with a reduction in neuroinflammation (Martín-Moreno et al., 2011; Cheng et al., 2014a).

The mechanisms underlying the neuroprotective effect of CBD against AD progression have not yet been fully elucidated. There is emerging evidence that the effect is mediated via PPARγ activation and subsequent inhibition of Nuclear Factor-Kappa B (Esposito et al., 2011; Scuderi et al., 2014). Nevertheless, mediation via alternative receptors has also been proposed, including the activation of TRPV1 and PI3K/Akt pathway (Libro et al., 2016). Future studies that determine the underlying mechanisms are warranted.

#### 4.1.4 Huntington’s disease

Huntington’s disease (HD) is an inherited disorder characterized by neuronal lesions in brain areas that help to control voluntary (intentional) movement, such as the cerebral cortex and the striatum (Rikani et al., 2014). As a result, HD patients develop uncontrollable movements (chorea) and abnormal body postures.

The neuroprotective properties of CBD make it a potential candidate in the treatment of HD. Indeed, in rats where striatal lesions were induced by 3-nitropropionic acid (3NP, an inhibitor of mitochondrial complex II), CBD reversed 3NP-induced reductions in GABA contents and reduced 3NP-induced striatal atrophy. The neuroprotective effects of CBD acted preferentially on striatal neurons that project to the substantia nigra and resulted from antioxidant and cannabinoid receptor-independent mechanisms (Sagredo et al., 2007).

To the best of our knowledge, there is only one clinical trial available that investigated the effects of CBD administration in patients suffering from HD. In this study, treatment with CBD (10 mg/kg/d for 6 weeks) in neuroleptic-free patients with HD did not show any effects on chorea severity or other therapeutic outcomes (Consroe et al., 1991). Other clinical trials investigating the effects of CBD on HD symptoms included THC-containing medicines, such as nabilone or Sativex (Curtis et al., 2009; López-Sendón Moreno et al., 2016). Besides the fact that these studies showed contradicting results, they did not allow us to determine the role of isolated CBD supplements in the treatment of HD.

Overall, evidence for the potential of CBD as a therapeutic agent to treat HD is lacking and future studies should aim to elucidate the isolated effects of CBD on HD symptoms.

#### 4.1.5 Parkinson’s disease

Parkinson’s disease (PD) is another neurodegenerative disease that is characterized by reduced dopamine levels as a result of dopaminergic neuron degeneration. Although the pathophysiology of PD is complicated, evidence suggests that α-synuclein (α-syn) aggregates play a significant role in the dopaminergic neurodegenerative process, leading to impairments of cellular function and oxidative stress (Pupyshev et al., 2018). The symptoms of PD include motor as well as non-motor signs such as depression, anxiety, apathy, sleep disorders, and psychosis (Poewe, 2008; Sveinbjornsdottir, 2016).

Currently, the dopamine replacement agent, I-DOPA, is often prescribed to treat PD. However, chronic use of I-DOPA is associated with (non-)motor complications including tardive dyskinesia (TD), a hyperkinetic movement disorder that is described by involuntary and repetitive movements (Hauser et al., 2022). Interestingly, CBD was able to reduce symptoms of orofacial dyskinesia induced by anti-dopaminergic drugs in mice (Sonego et al., 2018; Sonego et al., 2021) showing its potential as an add-on therapy in PD.

Furthermore, studies show that CBD ameliorates motor and cognitive impairments in animal models of PD and TD (da Cruz Guedes et al., 2023; Peres et al., 2016). More specifically, pretreatment with CBD (0.5 mg/kg) attenuated the increase in cataleptic behavior, oral movements and memory deficits, but not locomotor activity induced by reserpine administration in rats (Peres et al., 2016). In another study where *C. Elegans* were exposed to reserpine, CBD exposure recovered reserpine-induced alterations in locomotion rate/food-sensing behavior, attenuated morphologic alterations and dopaminergic neuron degeneration, and reduced human α-syn protein accumulation (da Cruz Guedes et al., 2023). These studies highlight the potential of CBD as a neuroprotector against PD.

However, clinical trials on the effects of CBD on PD symptoms show inconsistent results. Treatment with CBD for 10–15 days (titrated from 5 to 20–25 mg/kg/d) improved total and motor Movement Disorder Society Unified Parkinson Disease Rating Scale (UPDRS) scores in 10 PD patients (Leehey et al., 2020). These results were confirmed in an open-label pilot study where six patients received a flexible dose of CBD for 4 weeks (starting at 150 mg/d with weekly increases of ∼150 mg/d, depending on the clinical response). When CBD was added to their treatments, patients showed improvements in total UPDRS score (Zuardi et al., 2009). In contrast, a placebo-controlled double-blind RCT showed no changes in UPDRS score in participants who were treated with CBD (75 or 300 mg/d), although the scores on overall wellbeing and quality of life significantly improved (Chagas et al., 2014a).

As mentioned above, symptoms of PD also include non-motor signs such as anxiety, sleep disorders, and psychosis. Acute CBD supplementation (300 mg) was shown to reduce perceived feelings of anxiety and tremor amplitude in PD patients who underwent the simulated public speaking test (de Faria et al., 2020). Furthermore, psychotic symptoms were reduced in PD patients receiving a flexible dose of CBD (starting at 150 mg/d) for 4 weeks (Zuardi et al., 2009). Since the increased psychotic symptoms in PD patients are associated with the use of dopaminergic drugs, the authors hypothesized that the reduction in psychotic symptoms upon CBD treatment resulted from attenuated dopaminergic activity in brain areas related to psychotic symptoms. Regarding the effect of CBD on sleep disorders in PD, some discrepancies are present in the existing results. A case study of 4 PD patients suffering from rapid eye movement (REM) sleep behavior disorder (RBD) showed a reduction in the frequency of RBD events, including nightmares and active behavior during dreaming in all 4 patients after treatment with CBD (75 or 300 mg/d for 6 weeks) (Chagas et al., 2014b). However, these results were not confirmed in a placebo-controlled randomized trial in PD patients with Restless Legs Syndrome and RBD. In this study, treatment with CBD (doses gradually increasing from 75 to 300 mg for 12 weeks) did not result in improvements in subjective, nor in objective sleep quality measured by polysomnography, as compared to placebo (de Almeida et al., 2023).

Because of the different nature of these study designs (i.e., case studies and randomized controlled trials), the small sample sizes, and the different dosing schedules, it is difficult to draw solid conclusions on the effect of CBD on motor and non-motor symptoms in PD. Additional placebo-controlled trials are required to properly understand the role of CBD in this neurodegenerative disease.

### 4.2 Mental disorders

#### 4.2.1 Psychosis

Conventional treatment of psychosis is through drugs that are highly selective dopamine D2 receptor antagonists, such as haloperidol and amisulpride. Although effective, these treatments are often accompanied by severe side effects including deficits in motor control (Strange, 2001). Interestingly, animal models used for screening antipsychotic drugs, show that CBD treatment inhibited catalepsy or hyperlocomotion induced by apomorphine or ketamine (Zuardi et al., 1991; Moreira and Guimarães, 2005). These preclinical data indicate that CBD acts via different mechanisms, exhibiting a profile of atypical antipsychotic drugs. Therefore, CBD may be proposed as an alternative to classical drugs. The antipsychotic potential of CBD was demonstrated in schizophrenia (Zuardi et al., 1995; Leweke et al., 2012; McGuire et al., 2018) and PD (Zuardi et al., 2009).

While still speculative, current literature suggests that CBD may reduce psychotic symptoms via the inhibition of FAAH and subsequent increases in AEA levels. Indeed, in non-medicated acute schizophrenic patients, AEA cerebrospinal fluid concentrations showed a negative correlation with psychotic symptoms (Giuffrida et al., 2004). Interestingly, CBD (800 mg/d) treatment in acute schizophrenic patients increased AEA levels which was associated with reductions in psychotic symptoms (Leweke et al., 2012). Whereas these data present a convincing mechanism, alternative mechanisms of action such as the attenuation of increased glial reactivity (Gomes et al., 2015) or via parvalbumin-positive GABA neurons (Campos et al., 2017) cannot be excluded and further research is warranted.

A first individual case report of a female schizophrenia patient in which haloperidol treatment was terminated because of severe side effects, showed that treatment with CBD (1500 mg/d for 26 days) resulted in similar improvements in psychiatric symptoms without any side effects (Zuardi et al., 1995). Additionally, a clinical trial comparing amisulpride and CBD (800 mg/d for 4 weeks) treatment in acute schizophrenic patients showed that CBD was equally effective in treating clinical effects with fewer side effects compared to amisulpride (Leweke et al., 2012). Further evidence for the antipsychotic effects of CBD were provided in a trial where schizophrenia patients added CBD (1000 mg/d for 6 weeks) to their usual medication and perceived improvements in psychotic symptoms, as was supported by the treating clinicians’ impressions of improvement and illness severity (McGuire et al., 2018).

While these results are promising, it seems that higher doses up to 800 or 1000 mg/d are required to effectively treat psychotic symptoms, since chronic (6 weeks) or acute treatment with lower CBD doses (600 mg/d) did not improve symptoms in psychotic patients (Boggs et al., 2018) or cognitive impairments in schizophrenic patients (Hallak et al., 2010), respectively. Though, even these higher doses do not always seem to be effective, as it was shown that treatment with a high CBD dose of 1280 mg/d in three treatment-resistant schizophrenic patients only mildly improved symptoms of psychosis in one of the three patients after 35 days (Zuardi et al., 2006). It should be noted that the lack of effect in this case study may be explained by the severity of the disease state and does not necessarily exclude its effectiveness in non-resistant schizophrenic patients. Nevertheless, the application of CBD in psychotic patients requires further investigation regarding the optimal dosing schedule.

#### 4.2.2 Anxiety and fear

CBD treatment successfully induced anxiolytic-like effects in several animal models, including the elevated plus-maze (Guimarães et al., 1990; Resstel et al., 2009; Gomes et al., 2011; Hsiao et al., 2012), Y-maze (Mori et al., 2021), Vogel conflict test (Moreira et al., 2006; Gomes et al., 2011) and social interaction test (Almeida et al., 2013). Nevertheless, the mechanisms underlying the anxiolytic effects of CBD are not fully elucidated yet.

As previously mentioned, SPECT studies in healthy individuals and social anxiety disorder patients showed that CBD reduced anxiety and fear via its action on limbic and paralimbic brain areas (Crippa et al., 2004; Crippa et al., 2011). Furthermore, several animal models showed that the anxiolytic effects of CBD were blocked when combined with a 5-HT1A antagonist (Resstel et al., 2009; Gomes et al., 2011), suggesting mediation via this receptor. Indeed, 5-HT1A receptors are widely present in brain areas related to stress and anxiety and agonism of this receptor has been associated with anxiolytic responses (Rioja et al., 2004) whereas 5-HT1A-knockout mice showed anxiogenic-like behavior (Tsetsenis et al., 2007). In rats, pretreatment with a CB1 antagonist counteracted the suppressive effect of CBD on panic-like behavior induced by GABA_A_ receptor blockade (da Silva et al., 2015). Since CBD administration increases AEA levels, indirect agonism of CB1 might be an alternative mediating pathway through which CBD reduces anxiety levels. In addition, the anxiolytic effects of CBD in ischemic mice were prevented by antagonism at CB1 and 5-HT1A, as well as at CB2 and PPARγ (Mori et al., 2021), showing the complex signaling role of CBD.

Also, in human clinical trials, the anxiolytic effects of CBD have been determined. For example, acute administration with 300 mg CBD reduced anxiety induced by the simulated public speaking test (SPST) in healthy individuals (Zuardi et al., 1993; Zuardi et al., 2017; Linares et al., 2019) and PD patients (de Faria et al., 2020). Furthermore, patients suffering from social anxiety disorder showed higher levels of anxiety during the SPST compared to healthy individuals, but the administration of 600 mg CBD completely abolished these differences (Bergamaschi et al., 2011). Contrarily, administration of 600 mg CBD in healthy individuals without experimentally induced anxiety did not show any anxiolytic effect, suggesting that CBD is only able to reduce anxiety levels in case of stress or fearful situations (Martin-Santos et al., 2012). Furthermore, the dose is of relevance as it was shown that CBD presents an inverted U-shape in which intermediate doses seem to be effective, whereas low and high doses are not (Zuardi et al., 2017; Linares et al., 2019). Besides reducing anxiety levels, CBD also affected fear memory expression (Lee et al., 2017) via acute reduction in fear expression (Jurkus et al., 2016), enhancement of memory extinction (Bitencourt et al., 2008; Das et al., 2013) and disruption of memory reconsolidation (Stern et al., 2012; Gazarini et al., 2014; Stern et al., 2015). These effects may be of relevance for the treatment of phobias and post-traumatic stress disorder (PTSD).

#### 4.2.3 Depression

The antidepressant-like effects of CBD have been shown in several animal models including tail suspension (Schiavon et al., 2016), forced swimming (El-Alfy et al., 2010; Zanelati et al., 2010; Réus et al., 2011; Sales et al., 2019; Xu et al., 2019), the saccharine preference test (Shoval et al., 2016), and the novel object test (Shoval et al., 2016). It was repeatedly reported that CBD exerts its effect through 5-HT1A activation (Zanelati et al., 2010; Linge et al., 2016; Sartim et al., 2016) and increases in brain-derived neurotrophic factor (BDNF) levels in certain brain areas (Réus et al., 2011; Xu et al., 2019). Moreover, a recent study proposed that 5-HT1A activation by CBD, either directly or indirectly via elevated serotonin levels, may increase BDNF levels, which ultimately leads to mTOR activation and synaptogenesis (Sales et al., 2019). Nevertheless, not all animal studies replicate these findings as unaltered BDNF levels following CBD treatment have also been reported (Zanelati et al., 2010). These contradictory findings may be explained by the use of different animal species, different CBD doses, and different administration routes. In fact, the effectiveness of CBD seems highly dependent on the applied dose and studies suggest a U-shaped dose-response curve (Zanelati et al., 2010; Réus et al., 2011) as mentioned earlier. Additionally, doses that induce behavioral improvements do not always correspond to the doses that induce desired neurological adaptations (Réus et al., 2011; Schiavon et al., 2016). Future studies are warranted to properly understand the underlying mechanisms by which CBD improves symptoms of depression and to determine effective dose regimens.

Compared to the preclinical research, almost no studies investigated the effect of CBD on depression in humans. One case report showed that a patient suffering from neurofibromatosis type 1 perceived improvements in depressive symptoms when she switched from conventional antidepressants to CBD oil (Hegazy and Platnick, 2019). Given this scarcity, more clinical trials in humans are required to translate the preclinical antidepressant-like effects of CBD to therapeutic applications.

#### 4.2.4 Substance use disorders

An increasing number of studies focusing on the anti-addictive properties of CBD is emerging. Animal studies show promising effects of CBD in the context of alcohol, opioids, and methamphetamine use. Accordingly, a limited number of human studies showed a positive impact of CBD on nicotine, cannabis, and opioid use (Paulus et al., 2022).

There is a wide range of possible mechanisms via which CBD may be involved in the regulation of drug use and addiction (Navarrete et al., 2021). CBD may interact with the dopaminergic system, which plays an important role in addictive disorders. The release of dopamine after drug consumption induces drug-rewarding effects via the activation of dopamine D2 receptors (Trifilieff et al., 2013). CBD potentially inhibits the increased dopaminergic signaling induced by drug intake. Indeed, animal studies report that CBD treatment inhibited the amphetamine (Renard et al., 2016) and cocaine (Galaj et al., 2020) induced increases in neuronal dopamine activity. Additionally, these effects were associated with reductions in self-administration of cocaine (Galaj et al., 2020). Furthermore, reduced intake of ethanol by CBD was associated with lower gene expression of the μ-opioid receptor and tyrosine hydroxylase (Viudez-Martínez et al., 2018a), which is the rate-limiting enzyme in the biosynthesis of dopamine. The μ-opioid receptor is known to modulate dopamine transmission via inhibition of GABAergic interneurons (Spanagel et al., 1992). As such these findings propose an interaction between CBD and the opioid system.

Furthermore, CBD might exert its actions in substance use disorders via the serotonergic system. As previously mentioned, the agonistic actions of CBD at 5-HT1A have been widely acknowledged and seem to be involved in the anxiolytic and anti-depressant effects of CBD. In addition, the activation of 5-HT1A by CBD may also play a role in its mediation of the drug-rewarding process. In fact, mice subjected to oral ethanol self-administration showed lower ethanol intake when treated with a combination of CBD (20 mg/kg/d for 2 weeks) and Naltrexone (0.7 mg/kg/d for 2 weeks) (Viudez-Martínez et al., 2018b). These effects were absent when the treatment was combined with a 5-HT1A antagonist. Accordingly, a study using a self-administering paradigm of low cocaine doses in rats reported that the reductions in cocaine intake induced by CBD (20 or 40 mg/kg) were blocked by pretreatment with the 5-HT1A antagonist (Galaj et al., 2020). Interestingly, CBD was not able to reduce cocaine intake when self-administered cocaine doses were high, suggesting a limited capacity for CBD to counteract the reward process linked to cocaine consumption.

CBD might also be involved in the process of drug memory expression (Ren et al., 2009), consolidation (de Carvalho and Takahashi, 2017), and extinction (Parker et al., 2004), which plays an important role in addiction. Indeed, even though CBD treatment (5 and 20 mg/kg) was unable to reduce self-administrated heroin intake in rats, it did inhibit cue-induced drug-seeking behavior, a measure of cue-heroin memory expression (Ren et al., 2009). Additionally, several animal studies investigated the effect of CBD on drug memory consolidation and extinction using the drug-induced conditioned place preference paradigm, where increased time spent in the drug-paired place is associated with the rewarding properties of the drug. Using this paradigm, treatment with CBD impaired the preference for the place previously associated with amphetamine, morphine, or cocaine (Parker et al., 2004; de Carvalho and Takahashi, 2017). Whether CBD enhances extinction, impairs reconsolidation or influences both processes of drug memories is currently unclear and requires further investigation.

While the mechanisms mentioned above seem promising, most research in the field of the anti-addictive properties of CBD is based on animal models. The available evidence from clinical trials is scarce and human intervention trials are required to translate the preclinical findings towards therapeutic applications of CBD in substance use disorders.

### 4.3 Aspects of human behavior

#### 4.3.1 Sleep

CBD has been suggested as therapeutic strategy to improve sleep. The exact mechanisms via which CBD affects sleep are not yet fully understood, but different hypotheses are based on preclinical and clinical studies. On a biological level, it can be hypothesized that FAAH inhibition via CBD increases AEA levels, which might directly affect CB1-mediated NREM stability (Pava et al., 2016). However, it remains to be confirmed whether this CBD-FAAH-CB1 axis effectively mediates sleep. Alternatively, CBD can also directly act upon GABA_A_ receptors (Bakas et al., 2017), which play a beneficial role in sleep regulation (Gottesmann, 2002), e.g., reduced sleep latency and improved NREM sleep (Lancel, 1999). However, depending on the context (e.g., CBD levels and exposure time) and on the receptor subtype, CBD can act as a GABA_A_ receptor agonist or antagonist. Therefore, the CBD-GABA_A_ receptor axis also requires further investigation.

Despite biological mechanisms that might explain CBD-related improvements in sleep, it appears that CBD has no effect on sleep quality and quantity in subjects with an undisturbed and healthy sleep pattern (Linares et al., 2018). However, the calming effect of CBD on the CNS might reduce anxiety and promote relaxation (Shannon et al., 2019), via which CBD can indirectly improve sleep in people suffering from anxiety. In addition, some patient populations might also benefit from CBD use, since CBD increases sleeping time in insomnia patients (Carlini and Cunha, 1981) and improves REM sleep in Parkinson’s disease patients (Chagas et al., 2014b). It should be noted that some reports showed that CBD increased the wakefulness during sleep in young adults (Nicholson et al., 2004), and even decreased the REM sleep in rats (Murillo-Rodríguez et al., 2006; Murillo-Rodríguez et al., 2008). This alerting effect of CBD requires a well-planned timing and dosing of CBD if being used as a strategy to improve sleep, but this effect also holds promise for the use of CBD as a strategy to improve excessive daytime sleepiness (Nicholson et al., 2004).

#### 4.3.2 Motor control and cognition

Preclinical evidence indicates that acute and prolonged CBD use does not affect motor activity or spatial learning and recognition (Schleicher et al., 2019; Viudez-Martínez et al., 2019). However, in different (preclinical models of) diseases in which motor activity is compromised, CBD might improve some features of motor learning or attention.

Locomotion remained unaffected by CBD treatment in different preclinical models of hyper- and hypolocomotion, including Parkinson’s disease (hypo) (Peres et al., 2016), fragile X syndrome (hyper) (Zieba et al., 2019), and an acute neuroinflammatory model (hypo) (Florensa-Zanuy et al., 2021). Likewise, in a genetically-induced preclinical Alzheimer’s disease model, prolonged CBD treatment did not affect features of motor performance or sensorimotor gating (pole test, accelerod) (Coles et al., 2020). However, in a preclinical model of encephalopathy, CBD improved locomotion and cognition (Magen et al., 2009). An improvement in the memory deficit and object recognition memory was also observed in a Parkinson’s (Peres et al., 2016) and Alzheimer’s (Coles et al., 2020) disease model, respectively.

In schizophrenic patients, long-term but not acute CBD treatment (Hallak et al., 2010) improved the sustained attention assessed via the Continuous Performance Test (Leweke et al., 2021). Accordingly, CBD improved the attention lapse duration during a psychomotor vigilance test in healthy adults (Rudisill et al., 2023), whereas CBD had no effect on measures of attention during a vigilance test, and even exacerbated the impairing effect of THC in healthy rats (Moore et al., 2023). Altogether, CBD seems to have no consistent effect on motor performance in preclinical disease models. Some of the motor effects ascribed to CBD can be explained by its anxiolytic effect. It remains to be determined whether findings in preclinical models, often treated with supra-physiological doses of CBD, can be translated toward clinical populations.

#### 4.3.3 Memory

Several preclinical studies show the potential of CBD to restore memory impairments in a large variety of animal models that induce neurological dysfunction (Magen et al., 2009; Cassol et al., 2010; Magen et al., 2010; Avraham et al., 2011; Barichello et al., 2012; Fagherazzi et al., 2012; Pazos et al., 2012; Wright et al., 2013; Cheng et al., 2014a; Cheng et al., 2014b; Schiavon et al., 2014; Campos et al., 2015). A suggested mechanism via which CBD may improve or restore memory impairments involves its antioxidant properties. Research demonstrated that oxidative damage of nuclear as well as mitochondrial DNA in brain cells can have adverse effects on memory processing and retention during ageing (Kandlur et al., 2020). Animal models demonstrated that, by diminishing the risk of oxidative stress, CBD has the potential to safeguard brain cells (da Silva et al., 2018), thereby promoting the preservation of memory function. This notion has been supported in a study where treatment with CBD (2.5, 5, or 10 mg/kg daily for 9 days) in rats submitted to sepsis prevented memory alterations. In this study, the positive effects of CBD on memory were associated with a decrease in oxidative damage in the brain (Cassol et al., 2010).

Alternatively, the anti-inflammatory actions of CBD may play a role in restoring memory deficits. Moreover, in a murine model of cerebral malaria, treatment with CBD (30 mg/kg/d for 3 days, in addition to the traditional anti-malaria drug Artesunate) inhibited the increase in proinflammatory cytokines (TNFα and IL-6) in the hippocampus and prefrontal cortex and subsequently restored malaria-induced memory deficits (Campos et al., 2015). These results were replicated in a model of hepatic encephalopathy induced by bile-duct ligation in mice. Again, treatment with CBD (5 mg/kg/d for 4 weeks) restored impairments in cognition and locomotion and reduced the increase in hippocampal expression of the TNFα receptor 1 (Magen et al., 2009; Magen et al., 2010). These effects were mediated via 5-HT1A (Magen et al., 2010) and A2A adenosine (Magen et al., 2009) receptor activation, since treatment with their respective antagonists blocked the effects of CBD.

Interestingly, besides reducing neuroinflammation in these animal models, CBD treatment also restored hippocampal levels of BDNF (Magen et al., 2009; Magen et al., 2010; Campos et al., 2015). BDNF is a member of the neutrophin family and is highly involved in maintenance, growth, and survival of neurons. Given that previous studies show an association between increased BDNF levels and improved cognitive performance (Dincheva et al., 2012; Carlino et al., 2013), it seems plausible that CBD-induced increases in hippocampal BDNF explain, at least partly, the rescue of memory deficits in above mentioned models.

Lastly, it was hypothesized that CBD alters cerebral blood flow in brain regions that are involved in memory processing thereby influencing memory function. Moreover, in healthy human participants, acute supplementation with 600 mg CBD increased cerebral blood flow in the hippocampus (Bloomfield et al., 2020). Although higher resting hippocampal blood flow is associated with better memory performance (Heo et al., 2010), the current study was not able to show any effect of CBD supplementation on memory performance. The authors suggested that ceiling effects may have accounted for the lack of a relationship between hippocampal cerebral blood flow and memory task performance as the study population included healthy participants.

These results contrast with studies showing that CBD can restore or protect against episodic memory deficits induced by its psychotropic counterpart THC. More specifically, when healthy participants and cannabis users were administered THC (either 1.5 mg intravenously or 8 mg via inhalation) episodic memory and facial recognition performance were reduced. These effects were diminished when THC was combined with CBD (either 600 mg oral or 16 mg via inhalation) (Englund et al., 2013; Hindocha et al., 2015). However, these effects have not been confirmed in all studies and seem highly dependent on the dose, route of administration, and frequency of cannabis use of the study population (Morgan et al., 2018).

## 5 Future directions

### 5.1 Promising areas for future research

**FIGURE 2:**
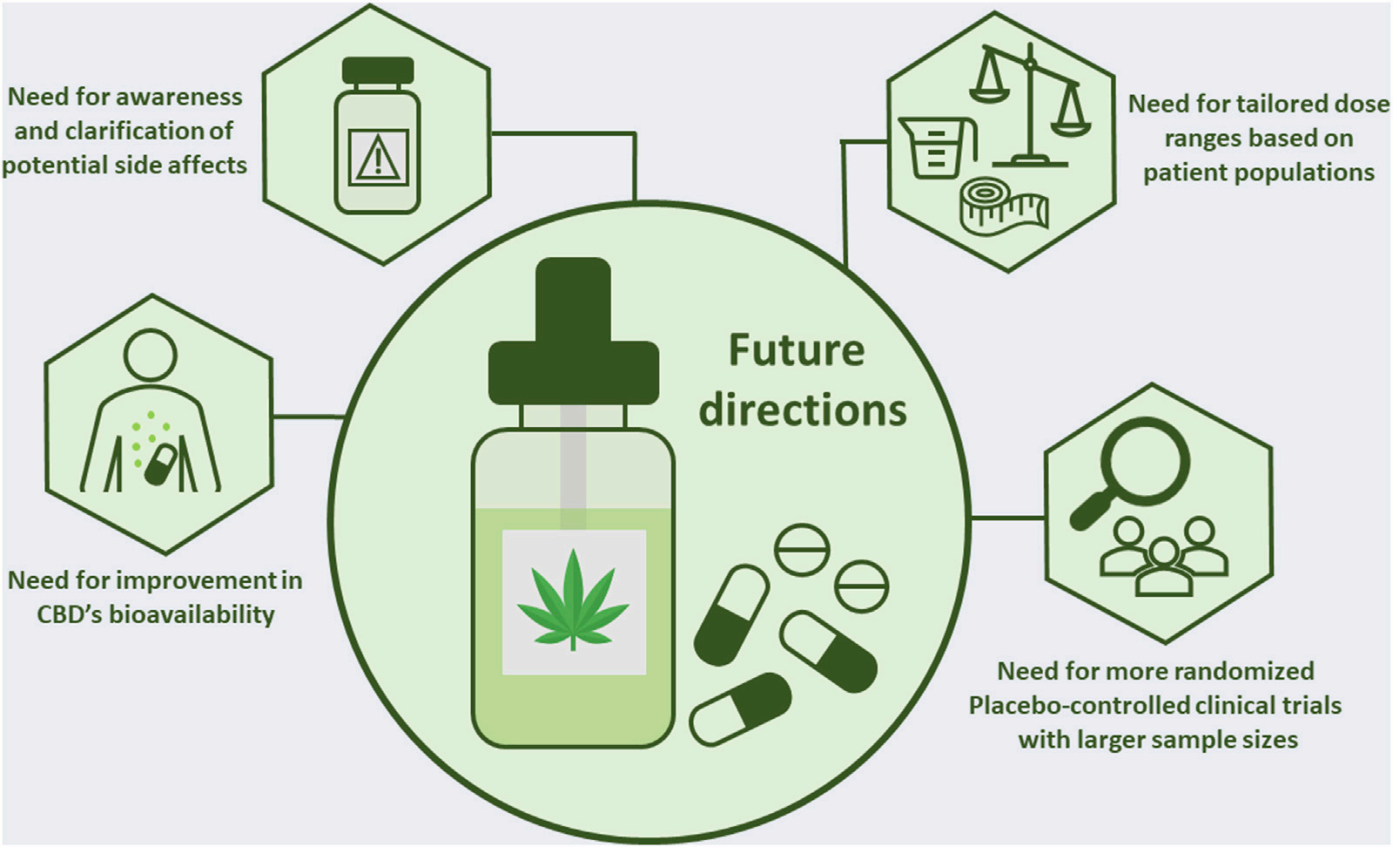
Future directions for research on CBD to confirm its use as a potential therapeutic agent.

The research on CBD is constantly evolving, and there are several promising areas of research that need to be further explored (Figure 2). One of the primary areas that future studies need to focus on is establishing a narrow range for CBD dosage, specifically tailored to the targeted disorder, consumer weight, ethnicity, and gender (Batalla et al., 2020; Legare et al., 2022). As demonstrated in the above sections, the current lack of an effective CBD dosage range results in mixed results. By leveraging brain imaging technology, researchers can more accurately determine the appropriate dosage range for specific disorders.

Another promising area of research is the use of CBD in the treatment of neurodegenerative disorders. While there have been a few human studies, the sample sizes have been small, the applied doses showed a large variability and control groups were often lacking. As a result, more placebo-controlled clinical trials are needed to clarify the effectiveness of CBD in the treatment of chronic pain, Alzheimer’s disease, Huntington’s disease, and Parkinson’s disease and to develop evidence-based guidelines for its use. Specifically for the management of chronic pain, there is a need to bridge the gap between what CBD users report in pain management and what is scientifically proven.

Finally, the effectiveness of CBD in treating mental disorders such as schizophrenia, anxiety and depression remains inconclusive. Also, the anti-addictive properties of CBD seem promising but human trials are scarce and future research should focus on the translation of the preclinical findings towards therapeutic applications of CBD.

### 5.2 Optimization of CBD’s therapeutic potential

The therapeutic potential of CBD is vast, and there are several ways in which this potential can be improved. One approach involves determining the active functional groups in the structure of the compound and manipulating it to improve its solubility without altering its therapeutic potential. This is particularly important because CBD has low bioavailability when taken orally, with only 6% of the compound being absorbed (Chayasirisobhon, 2020). This leads to a significant amount of wastage, which can be costly when the drug is commercialized. Linking CBD to a water-soluble end or a lipid compound without destroying its therapeutic ability could significantly increase its bioavailability and make it a more viable treatment option. In fact, co-ingesting CBD supplements with a high-fat meal was reported to significantly improve its bioavailability (Taylor et al., 2018; Birnbaum et al., 2019). Another approach to improving the therapeutic potential of CBD involves exploring different methods of administration. For example, aerosolized CBD has been shown to have a bioavailability of 31%, making it a potentially effective method of delivery (Stott et al., 2013; Chayasirisobhon, 2020). Finally, genetic engineering can be used to create strains of the cannabis plant with high CBD content, which is key to ensuring a consistent supply of CBD for production (Luo et al., 2019). This approach has the potential to significantly increase the availability of CBD and make it a more accessible treatment option for a wide range of conditions.

### 5.3 Ethical and regulatory considerations

The research field surrounding CBD remains quite volatile, and one should take certain issues into account. While CBD has been proven not to induce dependence, researchers must ensure the high purity of CBD used in their studies and be vigilant for possible contamination with THC. Also, they should carefully monitor any potential side effects that may arise (Chesney et al., 2020). Overall CBD is well tolerated with a very safe adverse event profile. Some recurring mild adverse events include somnolence, decreased appetite, and gastrointestinal symptoms (Lattanzi et al., 2021). Additionally, CBD is metabolized in the liver and influences enzymatic systems, as such drug-drug interactions between CBD and other medications that are metabolized in the liver may occur. In fact, clinical studies involving patients with the Lennox–Gastaut syndrome (Devinsky et al., 2018) or Dravet syndrome (Lattanzi et al., 2021) showed elevated serum transaminases when CBD treatment was combined with antiepileptic drugs. Even though most cases resolve either spontaneously or after dose reduction, these risks should be carefully considered before CBD treatment is prescribed. This is particularly important as the therapeutic potential of CBD continues to be explored for various medical conditions. By taking these precautions and ensuring that research is conducted in a safe and ethical manner, we can continue to advance our understanding of CBD and its potential applications in medicine, while also prioritizing the safety and wellbeing of study participants.

## 6 Conclusion

With significant research, CBD has the potential to meet the healthcare needs of different groups of neurological and psychiatric patients. Current studies indicate that CBD interacts with several GPCRs, such as CB1, CB2, GPR55, 5-HT1A, adenosine A2a, opioid and dopamine receptors, but it can also exert its action via the activation of ion channels (e.g., TRPV1 and voltage gated sodium and potassium channels) and via transcription factors (e.g., PPARγ), thereby targeting multiple pathways and mechanisms of action which may contribute to different therapeutic applications.

Animal and human brain disease models have been used to investigate the behavioral effects of CBD. Also, brain imaging methods such as fMRI and EEG have permitted the assessment of the CBD’s influence on brain functioning. A review of the available behavioral and neuroimaging data indicates that a drug concept is viable. Current research indicates that CBD is effective in managing epilepsy, especially when administered at an early stage, and can be used to manage anxiety and depression effectively. However, while the concept is there that CBD can be a solution for neurogenerative disorders, psychosis, and chronic pain, the research that has been done is still inconclusive and too often based on preclinical studies. Furthermore, more research is needed to define the right dosage. Ensuring that the dosage is respected is an actual challenge, as the medication might be abused.

## Figures and Tables

**Table:** 

**α-syn**	α-synuclein
**2-AG**	2-Arachidonoylglycerol
**3NP**	3-nitropropionic acid
**5-HT1A**	5-Hydroxytryptamine Receptor 1A
**5-HT1A**	Serotonin 1A Receptor
**AD**	Alzheimer’s Disease
**AEA**	Anandamide
**Akt**	Protein kinase B
**BDNF**	Brain-Derived Neurotrophic Factor
**CB1**	Cannabinoid Receptor Type 1
**CB2**	Cannabinoid Receptor Type 2
**CBD**	Cannabidiol
**CNS**	Central Nervous System
**ECS**	Endocannabinoid System
**EEG**	Electroencephalogram
**FAAH**	Fatty Acid Amide Hydrolase
**FDA**	Food and Drug Administration
**fMRI**	Functional Magnetic Resonance Imaging
**GABA**	Gamma-Aminobutyric Acid
**GABA**_**A**_	Gamma-Aminobutyric Acid Type A
**GPCR**	G Protein-Coupled Receptor
**GPR55**	G Protein-Coupled Receptor 55
**HD**	Huntington’s Disease
**I-DOPA**	L-Dihydroxyphenylalanine
**IEDs**	Interictal Epileptiform Discharges
**IL-6**	Interleukin 6
**KCNQ2**	potassium voltage-gated channel subfamily Q member 2
**(f)MRI**	Magnetic Resonance Imaging
**NAEs**	N-acylethanolamines
**OEA**	Oleoylethanolamide
**PD**	Parkinson’s Disease
**PEA**	Palmitoylethanolamide
**PET**	Positron Emission Tomography
**PPARα**	Peroxisome Proliferator-Activated Receptor Alpha
**PPARγ**	Peroxisome Proliferator-Activated Receptor GammaPI3K - Phosphoinositide 3-kinase
**PTSD**	Post-Traumatic Stress Disorder
**RBD**	REM Sleep Behavior Disorder
**RCT**	Randomized Controlled Trial
**(N) REM**	(Non-)Rapid Eye Movement
**SPECT**	Single-Photon Emission Computed Tomography
**SPST**	Simulated Public Speaking Test
**TD**	Tardive dyskinesia
**THC**	Tetrahydrocannabinol
**TNFα**	Tumor Necrosis Factor Alpha
**TRPV (1, 1a, 2)**	Transient Receptor Potential Vanilloid (1, 1a, 2)
**TRPM8**	Transient receptor potential melastin channel 8
**UPDRS**	Unified Parkinson Disease Rating Scale
