# Supplementary material for: Cannabidiol and brain function: current knowledge and future perspectives
Source: Front Pharmacol. 2024 Jan 15;14:1328885. doi: 10.3389/fphar.2023.1328885 (PMC10823027; doi:10.3389/fphar.2023.1328885)
Supplement: Supplementary file 1 [file Table1.DOCX]

**Table 1**. Imaging studies reporting acute effects of CBD on brain activity and connectivity.

| **Authors** | **Imaging technique** | **Participants** | **Age** | **CBD dose** | **Condition** | **Image analysis** | **Main findings** |
| --- | --- | --- | --- | --- | --- | --- | --- |
| **fMRI-studies** | | | | | | | |
| Bhattacharyya et al. (2009) | 1.5T fMRI | Healthy controls (n=15) | 26.7 (5.7) | 600mg (oral) | Verbal learning | Whole-brain | No effect |
| Bhattacharyya et al. (2012b) | 1.5T fMRI | Healthy controls (n=15) | 26.7 (5.7) | 600mg (oral) | Go/no-go | Whole-brain | ↓L medial prefrontal cortex |
| Bhattacharyya et al. (2015) | 1.5T fMRI | Healthy controls (n=15) | 26.7 (5.7) | 600mg (oral) | Go/no-go | Connectivity | ↓ R inferior frontal gyrus with R insula; L anterior lobe of cerebellum; L lingual gyrus; L thalamus; L dorsal striatum with L caudate nucleus body; L inferior frontal gyrus; L dorsal striatum with L anterior cingulate; L medial frontal gyrus; L posterior hippocampus with L parahippocampus; L posterior hippocampus with R parahippocampus; L posterior cingulate; L caudate tail |
| Bhattacharyya et al. (2018) | 3T fMRI | CHR-patients (n=33)  Healthy controls (n=19) | Patients:  25.4 (5.2)  Healthy:  23.9 (4.1) | 600mg (oral) | Verbal learning | Whole-brain | ↑ Pt activity in R inferior frontal gyrus, middle  frontal gyrus, insula; R precuneus, cuneus, lingual, middle occipital, fusiform gyri, cerebellum; L cerebellum, fusiform, lingual, inferior occipital gyri.  ↓ Pt activity in L parahippocampal gyrus, midbrain, cerebellum; L thalamus; L transverse temporal gyrus, superior temporal gyrus; L precentral, cingulate gyri, caudate body |
| Borgwardt et al. (2008) | 1.5T fMRI | Healthy controls (n=15) | 26.7 (5.7) | 600mg (oral) | Go/no-go | Whole-brain | ↓ L posterior insula, L superior temporal gyrus, L transverse temporal gyrus |
| Fusar-Poli et al. (2009) | 1.5T fMRI | Healthy controls (n=15) | 26.7 (5.7) | 600mg (oral) | Fearful faces | Whole-brain | Neutral faces: NS Intermediate fearful faces: ↓ BL posterior lobe cerebellum. Intensely fearful faces: ↓ L medial temporal region (amygdala and anterior parahippocampal gyrus), anterior and posterior cingulate gyri, R posterior lobe cerebellum |
| Fusar-Poli et al. (2010) | 1.5T fMRI | Healthy controls (n=15) | 26.7 (5.7) | 600mg (oral) | Fearful faces | Connectivity | ↓ connectivity of anterior cingulate cortex–amygdala |
| [Grimm et al. (2018)](#_bookmark34) | 3T fMRI | Healthy controls (n=16) | NR | 600mg (oral) | Resting state | Connectivity | ↑ connectivity of R putamen with R middle frontal gyrus, BL superior frontal gyrus/paracingulate gyrus, R frontal pole |
| O’Neill et al. (2020) | 3T fMRI | 13 Patients with psychotic disorders (n=13)  Healthy controls (n=19) | Patients:  27.7 (4.6)  Healthy:  23.9 (4.2) | 600mg (oral) | Verbal learning | ROI | Encoding: ↑ Pt activity in BL inferior frontal gyrus, L middle frontal gyrus  Recall: ↑ Pt activity in R middle -, R frontal gyrus, R parahippocampal gyrus.  ↑Pt connectivity between hippocampus and R caudate head, L caudate body, L putamen |
| Wilson et al. (2019) | 3T fMRI | CHR-patients (n=33)  Healthy controls (n=19) | Patients:  22.7 (5.1)  Healthy:  23.9 (4.2) | 600mg (oral) | Monetary  incentive  delay | ROI, whole-brain | ↑ Pt activity in L insula, parietal operculum; L  frontal operculum; L superior frontal gyrus |
| Winton-Brown et al. (2011) | 1.5T fMRI | Healthy controls (n=14) | 26.7 (5.7) | 600mg (oral) | Visual and auditory processing | Whole-brain | Auditory: BL temporal cortex, BL insula, BL parahippocampal gyri, BL hippocampi; L superior temporal gyrus, L insula, L posterior middle temporal  gyrus, L supramarginal gyrus  Visual: R (inferior, middle) occipital lobe, R lingual gyrus, R cerebellum, R cuneus |
| **SPECT-studies** | | | | | | | |
| Crippa et al. (2004) | 99mTc-ECD SPECT rCBF | Healthy controls (n=10) | 29.8 (5.1) | 400mg (oral) | Resting state | Whole-brain | ↑ L mediotemporal cortex (parahippocampus, fusiform gyrus). ↓ L. amygdala/hippocampus/hypothalamus, L posterior cingulate cortex |
| Crippa et al. (2011) | 99mTc-  ECD  SPECT  rCBF | Patients with with social anxiety (n=10) | 24.2 (3.7) | 400mg (oral) | Resting state | Whole-brain | ↓ L parahippocampal gyrus/hippocampus. ↑ R posterior cingulate gyrus |
| **MRS-studies** | | | | | | | |
| Pretzsch et al. (2019) | MRS | ASD-Patients (n=17),  Healthy controls (n=17) | Patients:  31.3 (9.9)  Healthy:  28.5 (6.6) | 600mg (oral) | Resting state | ROI | Hc and Pt: ↑ Glx in basal ganglia. ↓ Glx in dorsomedial prefrontal cortex  Hc: ↑ GABA+ in basal ganglia and dorsomedial prefrontal cortex |

ASD, autism spectrum disorder; BL, bilateral; CBD, cannabidiol; CHR, clinical high risk of psychosis; L, left; NR, not reported; R, right.
