# Supplementary material for: Cannabidiol and brain function: current knowledge and future perspectives
Source: Front Pharmacol. 2024 Jan 15;14:1328885. doi: 10.3389/fphar.2023.1328885 (PMC10823027; doi:10.3389/fphar.2023.1328885)
Supplement: Supplementary file 2 [file Table2.docx]

**Table 2**. (Pre)clinical studies reporting potential effects of CBD as therapeutic agent.

| **Authors** | **Study design** | **Animals/Participants** | **CBD treatment** | **Main findings** |
| --- | --- | --- | --- | --- |
| **Chronic pain** | | | | |
| **Preclinical studies** | | | | |
| Costa et al. (2007) | Intervention | Rat model of neuropathic (sciatic nerve chronic constriction) and inflammatory pain (complete Freund's adjuvant intraplantar injection) | 2.5–20 mg/kg/d (p.o.) to neuropathic pain group  20 mg/kg/d (p.o.) to inflammatory pain group  from day 7 to day 14 after the injury | Treatment with CBD reduced hyperalgesia to thermal and mechanical stimuli.  The anti-hyperalgesic effect of CBD was prevented by the vanilloid antagonist capsazepine, but not by cannabinoid receptor antagonists. |
| De Gregorio et al. (2019) | Intervention | Rats subjected to the spared nerve injury model | 5 mg/kg/d (s.c.) for 7 days | Treatment with CBD rescued impaired 5-HT neurotransmission under neuropathic pain conditions and reduced mechanical allodynia  Antiallodynic effects of CBD were fully prevented by the vanilloid antagonist capsazepine and partially prevented by 5-HT1A antagonist WAY 100635 |
| Jesus et al. (2019) | Intervention | streptozotocin-induced diabetic rats | acute or sub-chronical treatment (14 days) with 0.1, 0.3 or 3 mg/kg (i.p.) | Acute treatment with CBD (0.3 and 3 mg/kg) exerted a significant anti-allodynic effect. Which was prevented by the 5-HT1A antagonist WAY 100135, but not CB1 or CB2 receptor antagonists  Sub-chronic treatment with CBD (0.3 or 3 mg/kg) induced a sustained attenuation of the mechanical allodynia |
| Costa et al. (2004) | Intervention | Carrageenan-induced hyperalgesia in rats | 10mg/kg, acute | CBD treatment abolished hyperalgesia to a thermal stimulus.  This effect was reversed by the vanilloid antagonist capsazepine, but not by CB1 or CB2 antagonists |
| Belardo et al. (2019) | Intervention | Mice subjected to a weight drop mild TBI model | 30 µl, of CBD oil 10% (p.o.) day 1-14 and day 50- 60 | CBD restored chronic pain, anxious and aggressive behavior induced by TBI |
| Malvestio et al. (2021) | Intervention | Wistar rats subjected to chronic constriction injury of sciatic nerve | 30 nmol injected into prelimbic division of the medial prefrontal cortex, acute | CBD attenuated mechanical allodynia via CB1 and 5-HT1A dependent mechanisms |
| **Clinical studies** | | | | |
| Schilling et al. (2021) | Observational study / Survey | participants from pain management clinics (n=253) | none | most participants expressed a positive attitude about CBD products as a treatment option |
| Gulbransen et al. (2020) | Observational study | Patients grouped based on  1) non- cancer chronic pain symptoms (n=53)  2) neurological symptoms (n=21)  3) mental health-related symptoms (n=11)  4) cancer symptoms (n=24) | patient- titrated dosage ranging from 40-300mg/d  no control group | Patients with non-cancer pain and mental-health symptoms achieved improvements to patient- reported pain and depression and anxiety symptoms  CBD was well-tolerated |
| Cuñetti et al. (2018) | Intervention study / Open label intervention? | Kidney transplant patients with chronic pain who requested to receive CBD for pain treatment (n=7) | Initial dose of 100 mg/d with a progressive increase up to 300 mg/d (individualized)  No control group | CBD treatment resulted in optimal pain control in 2 patients, a partial response in 4 patients and no response in 1 patient.  CBD was well-tolerated, but individualization of treatment is recommended |
| Xu et al. (2020) | RCT | patients with symptomatic peripheral neuropathy (n=29) | transdermal application of CBD oil, 250 mg CBD/3 fl. oz | CBD treatment resulted in a reduction in intense pain, sharp pain, cold and itchy sensations |
| **Epilepsy** | | | | |
| **Preclinical studies** | | | | |
| Costa et al. (2022) | Intervention | Rat model of temporal lobe epilepsy | 120 mg/kg or 12 mg/kg twice a day for three days (subcutaneously) | CBD at 120 mg/kg abolished the seizures in 50% of rats and reduced total seizure duration and occurrence |
| Klein et al. (2017) | Intervention | Battery of multiple animal models of seizure | 0.01 ml/g in mice and 0.04 ml/10 g in rats. (i.p.) | CBD was protective in well-characterized rodent seizure models |
| Jones et al. (2012) | Intervention | Two seizure models (acute pilocarpine model of temporal lobe seizure and the penicillin model of partial seizure) in rats | 1, 10 and 100 mg/ kg (i.p.) acute | CBD reduced the incidence of the most severe seizures, reduced mortality and lowered the percentage of animals experiencing tonic–clonic seizures. |
| **Alzheimer’s Disease** | | | | |
| **Preclinical studies** | | | | |
| Scuderi et al. (2014) | Intervention | SHSY5Y^APP+^ Cells | 10^-9^–10^-6^ M | CBD treatment decreases amyloid precursor protein levels resulting in a decrease in Aβ production. CBD promoted cell survival by reducing apoptotic rate.  CBD effects were dependent PPARγ activation. |
| Libro et al. (2016) | Intervention | Mesenchymal stem cells derived from gingiva | 5 µM | treatment with CBD prevented the expression of proteins potentially involved in tau phosphorylation and Aβ production.  TRPV1 was able to mediate the modulatory effect of CBD via the PI3K/Akt pathway |
| Esposito et al. (2007) | Intervention | mouse model of AD-related neuroinflammation (inoculated with human Aβ) | 2.5 or 10 mg/kg/d (i.p.) for 7 days | CBD attenuated Aβ evoked neuroinflammatory responses |
| Esposito et al. (2011) | Intervention | Rat AD model (inoculated with human Aβ) | 10 mg/kg/d (i.p.) for 15 days | CBD reduced reactive gliosis, inhibited neuronal damage and stimulated hippocampal neurogenesis  These effects were PPARγ-dependent |
| Cheng et al. (2014) | Intervention | Transgenic mice-model of AD | 20 mg/kg/d (p.o.) for 8 months | CBD prevented the development of a social recognition deficit and showed a subtle reduction in neuroinflammation |
| Martín-Moreno et al. (2011) | Intervention | Mice-model of AD (intra- ventricularly injected with fibrillar Aβ) | 20 mg/kg (i.p.) daily during the first week, 3 days/week during the following 2 weeks | CBD prevented the Aβ-induced cognitive deficits shown by the animals in the Morris water maze  CBD treatment abolished Aβ-induced increase in IL-6 expression |
| **Huntington’s Disease** | | | | |
| **Preclinical studies** | | | | |
| Sagredo et al. (2007) | Intervention | rats intoxicated with 3-NP | 5 mg/kg/d for 5 days (i.p.) | CBD reversed the 3NP-induced reductions in GABA contents and provided neuroprotection against 3NP-induced striatal damage.  These effects were independent of CB1 or TRPV1 activation or adenosine signalling, but seemed to be exclusively based on the antioxidant properties of CBD. |
| **Clinical studies** | | | | |
| Consroe et al. (1991) | Crossover RCT | neuroleptic-free patients with Huntington's Disease (n=15) | 10 mg/kg/d (oral) for 6 weeks | CBD was neither symptomatically effective nor toxic, relative to placebo |
| López-Sendón Moreno et al. (2016) | Crossover RCT | Patients with Huntington's Disease (n=24) | 12 sprays/day of the Sativex oral spray, for 12 weeks  Providing  32.4mg THC/30 mg CBD /d | No significant symptomatic effects were detected  Sativex showed to be safe and well tolerated |
| Curtis et al. (2009) | Crossover RCT (pilot) | Patients with Huntington's Disease (n=44) | 1 or 2 mg Nabilone for 5 weeks (oral, capsules) | Treatment with Nabilone improved two neuropsychiatric outcome measures ([1]Behavior score on the Unified Huntington’s disease rating scale; [2]Neuropsychiatric inventory score)  Nabilone was well-tolerated |
| **Parkinson’s Disease** | | | | |
| **Preclinical studies** | | | | |
| Sonego et al. (2018) | Intervention | mice treated with haloperidol to induce tardive dyskinesia | 60 mg/kg/d (i.p.) for 21 days | CBD prevented haloperidol-induced orofacial dyskinesia by activating PPARγ receptors and attenuating neuroinflammatory changes in the striatum |
| Sonego et al. (2021) | Intervention | mice treated with haloperidol to induce tardive dyskinesia | 60 mg/kg/d (i.p.) for 7 days | CBD attenuated haloperidol- induced orofacial dyskinesia and improved non-motor symptoms associated with tardive dyskinesia by activating PPARγ receptors. |
| Da Cruz Guedes et al. (2023) | Intervention | C. elegans models exposed to reserpine | 3, 30 or 100 µM | CBD reversed the reserpine-induced locomotor alterations and acts as neuroprotector in dopaminergic neurons, reducing neurotoxicity and α-syn accumulation |
| Peres et al. (2016) | Intervention | Rats model of reserpine-induced tardive dyskinesia | 0.5 or 5 mg/kg/d for 4 days (i.p.) | CBD attenuated the increase in catalepsy behavior, oral movements and memory deficits, but not the decrease in locomotion induced by reserpine |
| **Clinical studies** | | | | |
| Leehey et al. (2020) | Open label intervention | Parkinson’s disease patients with substantial rest tremor (n=13) | titrated from 5 to 20–25mg/kg/day and maintained for 10–15 days | CBD improved total and motor Movement Disorder Society Unified Parkinson Disease Rating Scale scores in 10 patients |
| Zuardi et al. (2009) | Open label intervention | Parkinson’s disease patients who had psychosis (n=6) | flexible dose, starting from an oral dose of 150 mg/day) for 4 weeks | CBD resulted in a decrease in symptoms evaluated by the Brief Psychiatric Rating Scale and the Parkinson Psychosis Questionnaire |
| Chagas et al. (2014) | RCT | Parkinson’s disease patients without dementia or comorbid psychiatric conditions (n=21) | 75 or 300 mg/day for 6 weeks | CBD had no effect on Unified Parkinson Disease Rating Scale scores, but did improve quality of life measures |
| De Faria et al. (2020) | Crossover RCT | Parkinson’s disease patients (n=24) exposed to the Simulated Public Speaking Test | 300 mg, acute | CBD reduced anxiety and tremor amplitude induced by the Simulated Public Speaking Test |
| Chagas et al. (2014) | Case study | Parkinson’s disease patients with REM sleep behaviour disorder (n=4) | 75 or 300mg/d for 6 weeks | CBD reduced the frequency of RBD-related events without side effects. |
| De Almeida et al. (2023) | RCT | Parkinson’s disease patients with Restless Legs Syndrome and REM sleep behaviour disorder (n=18) | doses gradually increasing from 75-300mg for 12 weeks | CBD did not improve subjective, nor objective sleep quality measured by polysomnography |
| **Psychosis** | | | | |
| **Preclinical studies** | | | | |
| Zuardi et al. (1991) | Intervention | Rats exposed to Apomorphine-induced stereotypy | 15, 30, 60, 120, and 240 mg/kg (i.p.) acute | CBD attenuated the stereotypy induced by apomorphine and did not induce catalepsy even at the highest doses |
| Moreira et al. (2005) | Intervention | Mice receiving two psychotomimetic drugs, d-amphetamine or ketamine | 15–60 mg/kg (i.p.) acute | CBD inhibited hyperlocomotion without inducing catalepsy |
| Gomes et al. (2015) | Intervention | mice model of schizophrenia based | 60mg/kg (i.p.) for 22 days | CBD attenuated cognitive impairment and negative symptoms of schizophrenia and inhibited microglial activation |
| Almeida et al. (2013) | Intervention | Spontaneously Hypertensive Rats | 1, 5, 15, 30 or 60 mg/kg (i.p.), acute | CBD did induce antipsychotic effects |
| **Clinical studies** | | | | |
| McGuire et al. (2018) | RCT | patients with schizophrenia (n=88) | 1000 mg/day, for 6 weeks | CBD reduced levels of positive psychotic symptoms and was well-tolerated |
| Leweke et al. (2012) | RCT with comparative treatment | Patients diagnosed with schizophrenia or schizophreniform psychosis (n=39) | Gradual increase up to 800 mg/day for 4 weeks | CBD exerted clinically relevant antipsychotic effects and was well-tolerated |
| Zuardi et al. (1995) | Case report | female schizophrenia patient (n=1) | Gradual increase up to 1500 mg/d | CBD treatment improved psychotic symptoms and was well-tolerated |
| Zuardi et al. (2009) | Open label intervention | Parkinson’s disease patients who had psychosis (n=6) | flexible dose, starting from an oral dose of 150 mg/day for 4 weeks | CBD resulted in a decrease in symptoms evaluated by the Brief Psychiatric Rating Scale and the Parkinson Psychosis Questionnaire |
| Boggs et al. (2018) | RCT | Patients with chronic schizophrenia (n=36) | 600 mg/day for 6 weeks | CBD treatment was not associated with an improvement in psychotic symptoms  CBD was well-tolerated |
| Hallak et al. (1999) | RCT | schizophrenic patients (n=28) | 300 or 600mg, acute | CBD showed no beneficial effects on the performance of schizophrenic patients in the Stroop Color Word Test |
| Zuardi et al. (2006) | Open label intervention | treatment- resistant schizophrenia (n=3) | initial oral dose of 40mg reaching 1280mg/day for 30 days | Only one patient (out of 3) showed mild improvements in symptoms during CBD treatment  CBD was well-tolerated in all patients |
| **Anxiety** | | | | |
| **Preclinical studies** | | | | |
| Gomes et al. (2011) | Intervention | Rats exposed to models of anxiety (EPM and VCT) | 15, 30, or 60 nmol injected into the bed nucleus of the stria terminalis, acute | CBD exerted anxiolytic effects, which were blocked by 5-HT1A antagonism |
| Resstel et al. (2009) | Intervention | Rats submitted to restraint | 1, 10 or 20 mg kg^-1^ (i.p.) acute | CBD attenuated the anxiogenic response induced by restraint, which was blocked by 5-HT1A antagonism |
| Hsiao et al. (2012) | Intervention | Rats exposed to models of anxiety (EPM and open field) | 0.5 and 1.0μg injected into the central nucleus of amygdala, acute | CBD exerted anxiolytic effects |
| Guimaraes et al. (1990) | Intervention | Rats exposed to a model of anxiety (EPM) | 2.5, 5.0 and 10.0 (i.p.) mg/kg | CBD exerted anxiolytic effects |
| Mori et al. (2021) | Intervention | mice to bilateral common carotid artery occlusion and reperfusion | 10 mg/kg (i.p.) 0.5 hr before and 3, 24, and 48 hr after reperfusion | CBD prevented anxiety- like behavior. These effects were blocked by CB1, CB2, 5- HT1A, and PPAR- γ receptor antagonists. |
| Moreira et al. (2006) | Intervention | Rats exposed to a model of anxiety (VCT) | 2.5, 5 or 10 mg/kg (i.p.), acute | CBD exerted anxiolytic effects |
| Almeida et al. (2013) | Intervention | Spontaneously Hypertensive Rats | 1, 5, 15, 30 or 60 mg/kg (i.p.), acute | CBD (lowest dose) exerted anxiolytic effects as shown by increased social interactions |
| Jurkus et al. (2016) | Intervention | Rats exposed to contextual fear conditioning paradigm | 20 mg/kg (i.p), acute | CBD decreased auditory fear memory expression without affecting its extinction |
| Bitencourt et al. (2008) | Intervention | Rats exposed to contextual fear conditioning paradigm | 2.0 µg/µl (i.c.v.), acute | CBD facilitated extinction of contextual fear memory |
| Gazarini et al. (2014) | Intervention | Rats exposed to contextual fear-conditioning paradigm | 10 mg/kg (i.p.), acute | cannabidiol significantly disrupted the reconsolidation process |
| Stern et al. (2012) | intervention | Rats exposed to contextual fear-conditioning paradigm | 3–30 mg/kg (i.p.) | CBD blocks reconsolidation, rather than facilitating extinction following contextual fear-conditioning |
| Stern et al. (2015) | Intervention | Rats exposed to contextual fear-conditioning paradigm | 0.1 mg/kg THC + 1.0 mg/kg CBD | THC and CBD attenuated fear memory maintenance in an additive fashion |
| Da Silva et al. (2015) | Intervention | Rats subjected to GABA_A_ receptor blockade to induce panic-like responses | 5.0 μg/0.2 μl injections into the substantia nigra, pars reticulata | CBD reduced defensive alertness and explosive escape behaviour |
| **Clinical studies** | | | | |
| Zuardi et al. (2017) | RCT | Healthy volunteers subjected to a test of public speaking in a real situation (n=60) | 100, 300, and 900 mg, acute | CBD induced anxiolytic effects with a dose-dependent inverted U-shaped curve, where only 300 mg showed to be effective |
| Zuardi et al. (1993) | RCT | Healthy volunteers subjected to a simulated public speaking test (n=40) | 300 mg, acute | CBD induced anxiolytic effects |
| Linares et al. (2019) | RCT | Healthy volunteers subjected to a simulated public speaking test (n=57) | 150, 300, and 600 mg, acute | CBD induced anxiolytic effects with a dose-dependent inverted U-shaped curve, where only 300 mg showed to be effective |
| Carlini et al. (1981) | Crossover RCT | Insomniac volunteers (n=15) | 160 mg, acute | CBD increased time asleep and reduced dream recall |
| De Faria et al. (2020) | Crossover RCT | Parkinson’s disease patients (n=24) exposed to the SPST | 300 mg, acute | CBD reduced anxiety and tremor amplitude induced by the SPST |
| Bergamischi et al. (2011) | RCT | healthy control patients and treatment-naïve Social Anxiety Disorder patients subjected to the SPST | 600 mg, acute | CBD diminished the increase in anxiety induced by the SPST on Social Anxiety Disorder patients, resulting in a similar response as the healthy controls |
| Martin-Santos et al. (2012) | Crossover RCT | Healthy male subjects, not exposed to any intervention besides supplementation | 600 mg, acute | CBD did not induce any symptomatic or physiological changes compared to placebo |
| Das et al. (2013) | RCT | Healthy volunteers (n=48) exposed to a Pavlovian fear-conditioning paradigm | 32 mg either following before or after extinction | CBD enhanced consolidation of extinction learning suggesting a role for CBD as an adjunct to extinction-based therapies for anxiety disorders. |
| **Depression** | | | | |
| **Preclinical studies** | | | | |
| Schiavon et al. (2016) | Intervention | Mice subjected to the TST | 3 mg/kg (i.p.), acute | CBD induced anti-depressant effects as shown by reduced immobility time during the TST |
| Xu et al. (2019) | Intervention | Mice subjected to a chronic mild stress model | 100 mg/kg (p.o.) or 10 mg/kg CBD (i.v.), acute | CBD elicited significant antidepressant-like behavioral effects in forced swim test |
| Zanelati et al. (2010) | Intervention | Mice subjected to the FST | 30 mg/kg (i.p.), acute | CBD induces antidepressant-like effects during the FST. These effects were blocked by the 5-HT1A antagonist |
| El-Alfy et al. (2010) | Intervention | Mice subjected to the FST and TST | 200 mg/kg (i.p.), acute | CBD induces antidepressant-like effects during the FST, but not TST.  No changes in BDNF levels |
| Réus et al. (2011) | Intervention | Mice subjected to the FST | 30 mg/kg (i.p.), acute  15 mg/kg (i.p.) for 14 days | Acute and chronic treatment with CBD induced antidepressant effects during the FST.  Chronic treatment increased BDNF levels in the amygadal. |
| Sales et al. (2019) | Intervention | Mice subjected to the FST | 10 mg/kg (i.p.) acute or for 7 days | CBD induces fast and sustained antidepressant-like effect  The acute antidepressant effects of CBD were associated with increased BDNF levels |
| Shoval et al. (2016) | Intervention | ‘Depressive-like’ Wistar-Kyoto rat subjected to the SPT, EPM and NOE | 15, 30 and 45 mg/kg (p.o.) | Depending on dose, CBD showed anti-depressant effects in the SPT, and improved the motivation to explore in the NOE. |
| Linge et al. (2016) | Intervention | Mice subjected to the olfactory bulbectomy model of depression | 50 mg/kg (i.p.) acute  10 mg/kg/d (i.p) for 14 days | CBD exerts fast and maintained antidepressant-like effects, which is prevented by 5-HT1A receptor blockade |
| Sartim et al. (2016) | Intervention | Rats subjected to the FST and OFT | 10–60 nmol/side intra- infralimbic or -prelimbic, acute | CBD into the ventral medial prefrontal cortex induces antidepressant-like effects, which were blocked by the 5-HT1A antagonist |
| **Clinical studies** | | | | |
| Hegazy et al. (2019) | Case report | Female patient with Neurofibromatosis type 1 (n=1) | sublingual CBD oil at 4 mg/d titrated upwards to 10 mg/d for 3 months | CBD treatment resulted in improvements in pain scores and reduced depressive symptoms |
| **Substance use disorder** | | | | |
| **Preclinical studies** | | | | |
| Renard et al. (2016) | Intervention | Rats subjected to amphetamine-induced sensitization | 0.50μl injected into the nucleusaccumbens shell for 5 days | CBD attenuated amphetamine-induced dopaminergic neuronal activity within the ventral tegmental area |
| Galaj et al. (2020) | Intervention | Rats subjected to cocaine self-administration | 10-40 mg/kg (i.p.), acute | CBD cocaine-induced increases in extracellular dopamine in the nucleus accumbens, and inhibited cocaine self-administration (only at low doses of cocaine). These effects were dependent on CB2, 5-HT1A and TRPV1 receptor mechanisms |
| Viudez-Martínez et al. (2018) | Intervention | Mice subjected to the two-bottle choice paradigm and ethanol self-administration | 30, 60 and 120 mg/kg/day (i.p.), for 5 days (plus naltrexone) | CBD reduced ethanol consumption and preference in the two-bottle choice and reduced the relative gene expression of tyrosine hydroxylase |
| Ren et al. (2009) | Intervention | Rats subjected to heroin self-administration | 5 and 20 mg/kg (i.p.), acute | CBD did not alter heroin self-administration but did inhibit cue-Induced Heroin Seeking |
| de Carvalho et al. (2017) | Intervention | Rats subjected to the place preference conditioning paradigm | 10 mg/kg (s.c), acute | CBD disrupted the reconsolidation of contextual drug-related memories |
| Parker et al. (2004) | Intervention | Rats subjected to the place preference conditioning paradigm | 5 mg/kg (i.p.), acute | CBD potentiated the extinction of both cocaine- and amphetamine-induced conditioned place preference learning |
| **Sleep** | | | | |
| **Preclinical studies** | | | | |
| Murillo-Rodríguez et al. (2006) | Intervention | Healthy rats | 10μg/5μl (i.c.v.), acute | CBD increased wakefulness and decreased REM sleep |
| Murillo-Rodríguez et al. (2008) | Intervention | Healthy rats | 10 or 20 μg/μl into the lateral hypothalamus or dorsal raphe nuclei, acute | CBD enhanced wakefulness and decreased slow wave sleep and REM sleep |
| **Clinical studies** | | | | |
| Linares et al. (2018) | Crossover RCT | Healthy volunteers without sleep difficulties (n=27) | 300mg, acute | CBD did not alter normal sleep architecture |
| Shannon et al. (2019) | retrospective case series | Patients from a psychiatric clinic (n=72) | 25 mg/d for 3 months  (A handful of patients were given 50 mg/d or 75 mg/d) | CBD reduced anxiety levels and improved self-perceived sleep quality |
| Chagas et al. (2014) | Case study | Parkinson’s disease patients with REM sleep behaviour disorder (n=4) | 75 or 300mg/d for 6 weeks | CBD reduced the frequency of RBD-related events without side effects. |
| Nicholson et al. (2004) | Crossover RCT | Healthy volunteers (n=8) | 5 mg THC + 5 mg CBD or 15 mg THC + 15 mg CBD via an oromucosal spray | concomitant administration of THC and CBD reduced stage 3 sleep and increased awake time |
| **Motor control and cognition** | | | | |
| **Preclinical studies** | | | | |
| Viudez-Martínez et al. (2018) | Intervention | Mice subjected to the OFT | 30 mg/kg/12 h (i.p.) for 6 days | CBD did not affect motor activity |
| Schleicher et al. (2019) | Intervention | Mice subjected to a battery of behavioral tests | 20 mg/kg (i.p.) for 6 weeks | CBD did not affect motor performance or spatial learning |
| Peres et al. (2016) | Intervention | Rats model of reserpine-induced tardive dyskinesia | 0.5 or 5 mg/kg/d for 4 days (i.p.) | CBD attenuated the increase in catalepsy behavior, oral movements and memory deficits, but not the decrease in locomotion induced by reserpine |
| Zieba et al. (2019) | Intervention | Mice model of Fragile X Syndrome subjected to behavioral tasks | 5 or 20 mg/kg (i.p.), acute | CBD had no impact on locomotion |
| Florensa-Zanuy et al. (2021) | Intervention | Mice subjected to LPS-induced neuroinflammation | 30 mg/kg (i.p.), acute | CBD had no impact on locomotion |
| Coles et al. (2020) | Intervention | transgenic mouse model for AD | 5 mg/kg/d (i.p.), for 3 weeks | CBD treatment did not affect the hyperlocomotion or motor impairments, but CBD did reverse the deficits in object recognition and delayed spatial learning in transgenic mice. |
| Magen et al. (2009) | Intervention | mice subjected to bile duct ligation | 5 mg/kg (i.p.), for 4 weeks | CBD improved impairments in cognitive and locomotor function and restored expression of the TNFα receptor 1. Effects were mediated via the A2A adenosine receptor |
| Moore et al. (2023) | Intervention | Rats subjected to the psychomotor Vigilance Test | 1-100 mg/kg CBD or 1-17.6 mg/kg THC and combinations (p.o.) | CBD alone had no effect on Sustained Attention, but further exacerbated the disruptions induced by THC |
| **Clinical studies** | | | | |
| Leweke et al. (2021) | placebo-controlled randomized trial | acute paranoid schizophrenic patients (n=42) | up to 800 mg/day for 4 weeks | CBD improved sustained attention and visuomotor coordination |
| Hallak et al. (1999) | RCT | schizophrenic patients (n=28) | 300 or 600mg, acute | CBD showed no beneficial effects on the performance of schizophrenic patients in the Stroop Color Word Test |
| Rudisill et al. (2023) | RCT | Healthy volunteers (n=40) | 300 mg, acute | CBD increased attention lapse duration in the Psychomotor Vigilance Test and improved auditory reaction time during the Simple Reaction Time Test |
| **Memory** | | | | |
| **Preclinical studies** | | | | |
| Cheng et al. (2014) | Intervention | Transgenic mice-model of AD | 20 mg/kg/d (p.o.) for 8 months | CBD prevented the development of a social recognition deficit and showed a subtle reduction in neuroinflammation |
| Magen et al. (2010) | Intervention | mice subjected to bile duct ligation | 5 mg/kg (i.p.), for 4 weeks | CBD improved impairments in cognitive and locomotor function and restored expression of the TNFα receptor 1 and BDNF genes. Effects were mediated via the 5-HT1A-receptor |
| Magen et al. (2009) | Intervention | mice subjected to bile duct ligation | 5 mg/kg (i.p.), for 4 weeks | CBD improved impairments in cognitive and locomotor function and restored expression of the TNFα receptor 1 and BDNF genes. Effects were mediated via A2A adenosine receptor |
| Cassol et al. (2010) | Intervention | Rats subjected to cecal ligation and perforation | 2.5, 5, or 10 mg/kg/d (i.p.) for 9 days | CBD reduced oxidative damage and prevented memory alterations observed in septic rats |
| Fagherazzi et al. (2012) | Intervention | Rats subjected to a model of cognitive impairment (iron overload) | 5 or 10.0 mg/kg (i.p.), acute or for 14 days | Acute and chronic CBD administration rescued memory in iron-overloaded animals |
| Campos et al. (2015) | Intervention | Mice model of Cerebral malaria | 30 mg/kg/d (i.p.) for 7 days | CBD prevented memory deficits, decreased levels of proinflammatory cytokines and increased hippocampal levels of BDNF |
| Barichello et al. (2012) | Intervention | Rats submitted to pneumococcal meningitis | 10 mg/kg/d (i.p.) for 9 days | CBD reduced levels of TNFα and prevented memory impairments |
| Avraham et al. (2011) | Intervention | Mice model of hepatic encephalopathy | 5 mg/kg (i.p.), acute | CBD restored impairments in neurological and cognitive functions |
| Cheng et al. (2014) | Intervention | transgenic mouse model for AD | 20 mg/kg/d (i.p) for 3 weeks | CBD reversed the impairments in in social recognition and novel object recognition |
| Wright et al. (2013) | Intervention | Healthy rhesus monkeys challenged with THC (0.2, 0.5 mg/kg i.m.) | 0.5 mg/kg CBD (i.m.), acute | CBD opposed the cognitive impairing effects of THC on paired-associates learning and a bimanual motor task |
| Pazos et al. (2012) | Intervention | rats exposed to hypoxia-ischemia injury | 1 mg/kg CBD (s.c.), acute | CBD treatment prevented the memory deficit caused by the hypoxia-ischemia insult |
| Schiavon et al. (2014) | Intervention | mice subjected to bilateral common carotid artery occlusion | 3, 10 or 30 mg/kg (i.p.) before and 3, 24, and 48 h after surgery | CBD improved spatial learning performance |
| Da Silva et al. (2018) | Intervention | Rats subjected to a model of cognitive impairment (iron overload) | 10 mg/kg/d (i.p.) for 14 days | CBD rescued mitochondrial ferritin and epigenetic modulation of mtDNA, and restored succinate dehydrogenase activity in iron-treated rats. |
| **Clinical studies** | | | | |
| Bloomfield et al. (2020) | Crossover RCT | Healthy volunteers (n=15) | 600 mg, acute | CBD increased cerebral blood flow in the hippocampus, did not affect memory task performance |
| Hindocha et al. (2015) | Crossover RCT | light and heavy cannabis users (n=48) | THC (8 mg), CBD (16 mg), THC +CBD (8 mg+16 mg) by inhalation, acute | CBD improved emotional facial affect recognition and attenuates the impairment induced by THC |
| Englund et al. (2013) | RCT | Healthy volunteers (n=48) | 600 mg CBD (oral) prior to 1.5 mg THC (i.v.) | pre-treatment with CBD protected episodic memory from the impact of THC |
| Morgan et al. (2018) | Crossover RCT | Cannabis users (n=48) | 8 mgTHC, 16 mg CBD or 8 mg THC + 16 mg CBD by inhalation, acute | CBD did not attenuate the acute psychotic and memory impairing effects of vaporised THC |

*A non-exhaustive overview of the literature concerning the effects of CBD on a large variety of neurological and mental disorders and aspects of human behavior. 3-NP, 3-nitropropionate ; AD, Alzheimer’s Disease; Aβ, Amyloid beta; BDNF, brain-derived neurotrophic factor; CBD, Cannabidiol; EPM, Elevated Plus Maze; FST, forced swimming test; i.c.v., Intracerebroventricular; i.m., intramuscular; i.p., intraperitoneal; LPS, Lipopolysaccharide; mtDNA, mitochondrial DNA; NOE, novel object exploration test; OFT, Open field test; p.o., Per oral; RCT, randomized control trial; REM sleep, rapid eye movement sleep; s.c.,subcutaneous; SPST, Simulated public speaking test; SPT, saccharin preference test; TBI, Traumatic Brain Injury; THC, Tetrahydrocannabinol; TNFα, Tumor Necrosis Factor Alpha; TST, the tail suspension test; VCT, Vogel conflict test;*
